# Supplementary figures and images for: Tmem263 deletion disrupts the GH/IGF-1 axis and causes dwarfism and impairs skeletal acquisition
Source: eLife. 2024 Jan 19;12:RP90949. doi: 10.7554/eLife.90949 (PMC10945605; doi:10.7554/eLife.90949)

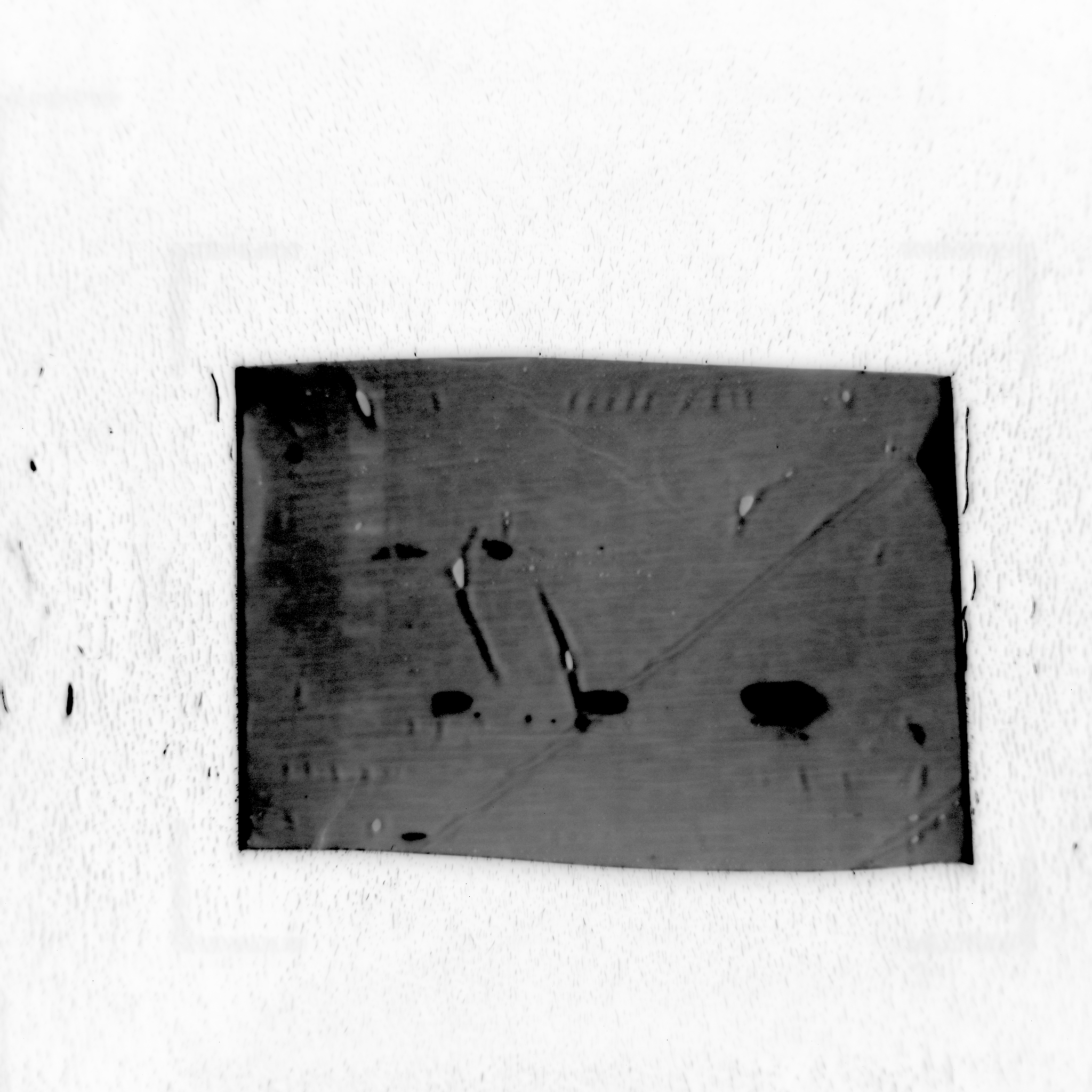

Supplement: Figure 1—source data 1. — Two close molecular markers are noted. FLAG-tagged TMEM263 at the predicted molecular weight is marked. The cropped region used for the main figure image is marked with a dotted box. This membrane was probed with the anti-FLAG antibody. Bottom left – The same membrane as shown above, but overexposed and imaged with the light channel to show the entire membrane outline. The anti-FLAG antibody is very clean and therefore the membrane boarder is hard to see otherwise. Top right – Original uncropped membrane from imager showing blue channel only as a black and white image. Two close molecular markers are noted. FLAG-tagged TMEM263 at the predicted molecular weight is marked. The cropped region used for the main figure image is marked with a dotted box. This membrane was probed with the anti-FLAG antibody. Bottom left – The same membrane as shown above, but overexposed and imaged with the light channel to show the entire membrane outline. The anti-TMEM263 antibody is fairly clean and therefore the membrane boarder is hard to see otherwise. [file elife-90949-fig1-data1.zip › Figure 1-source data 1/Figure 1-source data 1 - original uncropped - light image overlay on overexposed membrane - anti-TMEM263.tif]

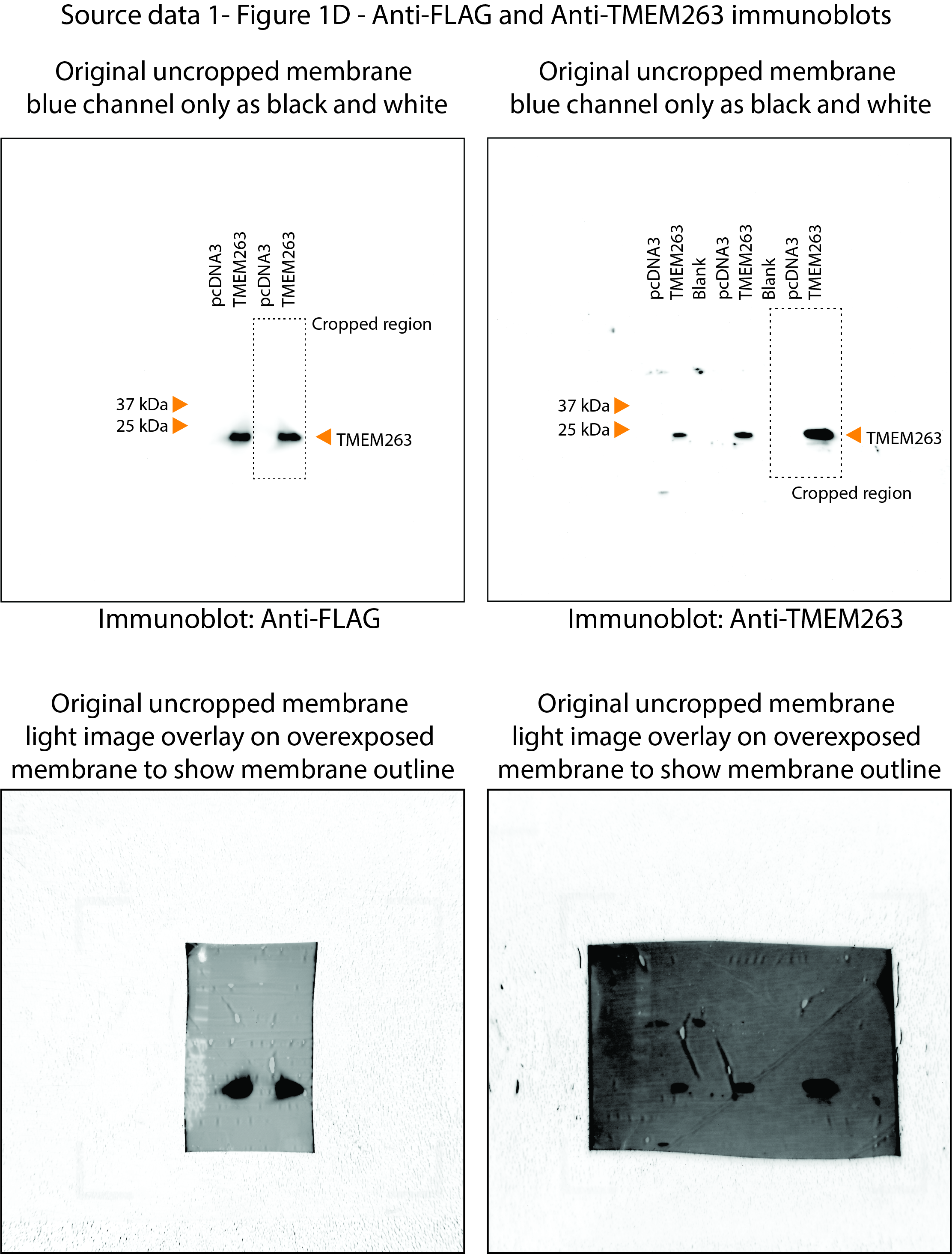

Supplement: Figure 1—source data 1. — Two close molecular markers are noted. FLAG-tagged TMEM263 at the predicted molecular weight is marked. The cropped region used for the main figure image is marked with a dotted box. This membrane was probed with the anti-FLAG antibody. Bottom left – The same membrane as shown above, but overexposed and imaged with the light channel to show the entire membrane outline. The anti-FLAG antibody is very clean and therefore the membrane boarder is hard to see otherwise. Top right – Original uncropped membrane from imager showing blue channel only as a black and white image. Two close molecular markers are noted. FLAG-tagged TMEM263 at the predicted molecular weight is marked. The cropped region used for the main figure image is marked with a dotted box. This membrane was probed with the anti-FLAG antibody. Bottom left – The same membrane as shown above, but overexposed and imaged with the light channel to show the entire membrane outline. The anti-TMEM263 antibody is fairly clean and therefore the membrane boarder is hard to see otherwise. [file elife-90949-fig1-data1.zip › Figure 1-source data 1/Figure 1-source data 1.tif]

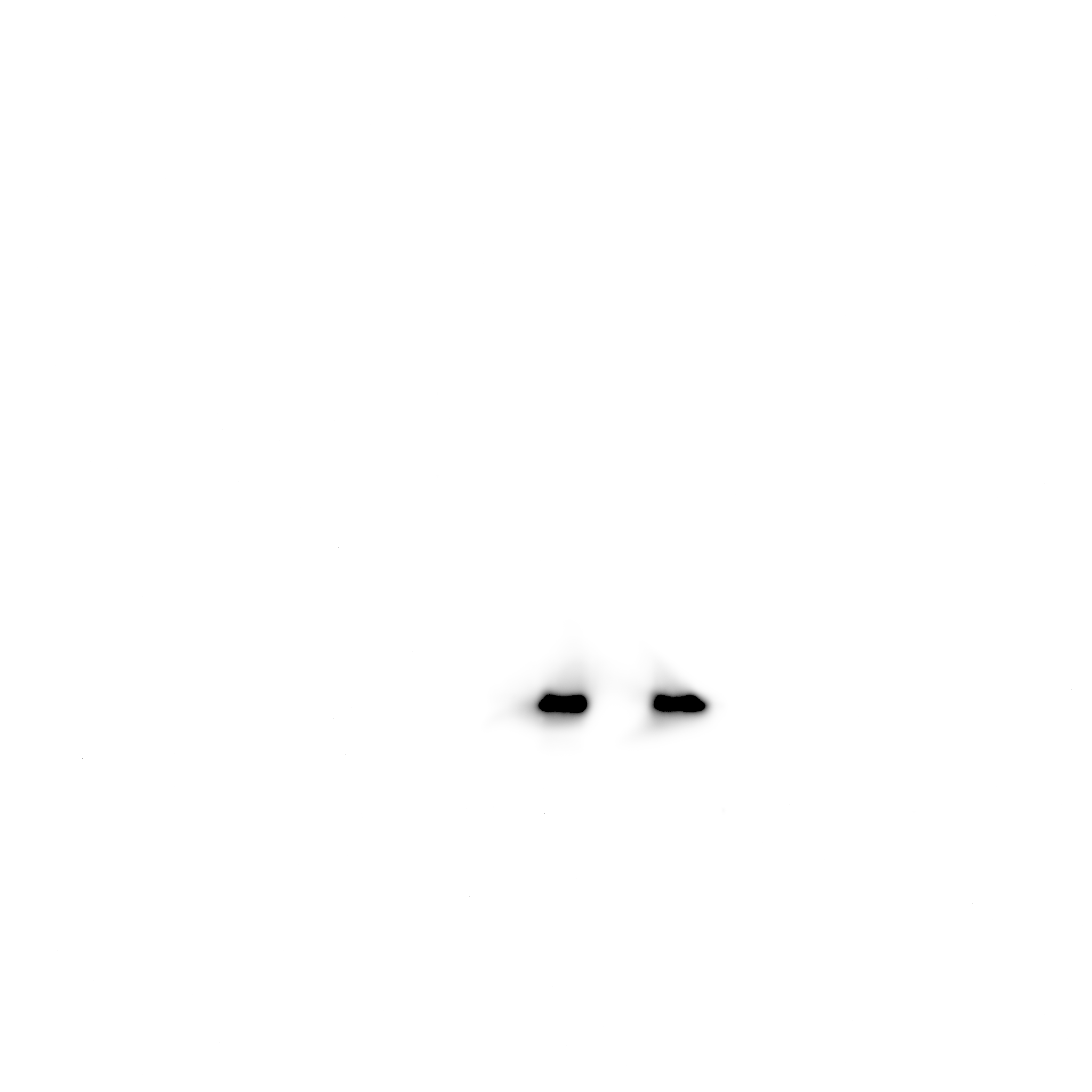

Supplement: Figure 1—source data 1. — Two close molecular markers are noted. FLAG-tagged TMEM263 at the predicted molecular weight is marked. The cropped region used for the main figure image is marked with a dotted box. This membrane was probed with the anti-FLAG antibody. Bottom left – The same membrane as shown above, but overexposed and imaged with the light channel to show the entire membrane outline. The anti-FLAG antibody is very clean and therefore the membrane boarder is hard to see otherwise. Top right – Original uncropped membrane from imager showing blue channel only as a black and white image. Two close molecular markers are noted. FLAG-tagged TMEM263 at the predicted molecular weight is marked. The cropped region used for the main figure image is marked with a dotted box. This membrane was probed with the anti-FLAG antibody. Bottom left – The same membrane as shown above, but overexposed and imaged with the light channel to show the entire membrane outline. The anti-TMEM263 antibody is fairly clean and therefore the membrane boarder is hard to see otherwise. [file elife-90949-fig1-data1.zip › Figure 1-source data 1/Figure 1-source data 1- original uncropped - immunoblot of anti-FLAG.tif]

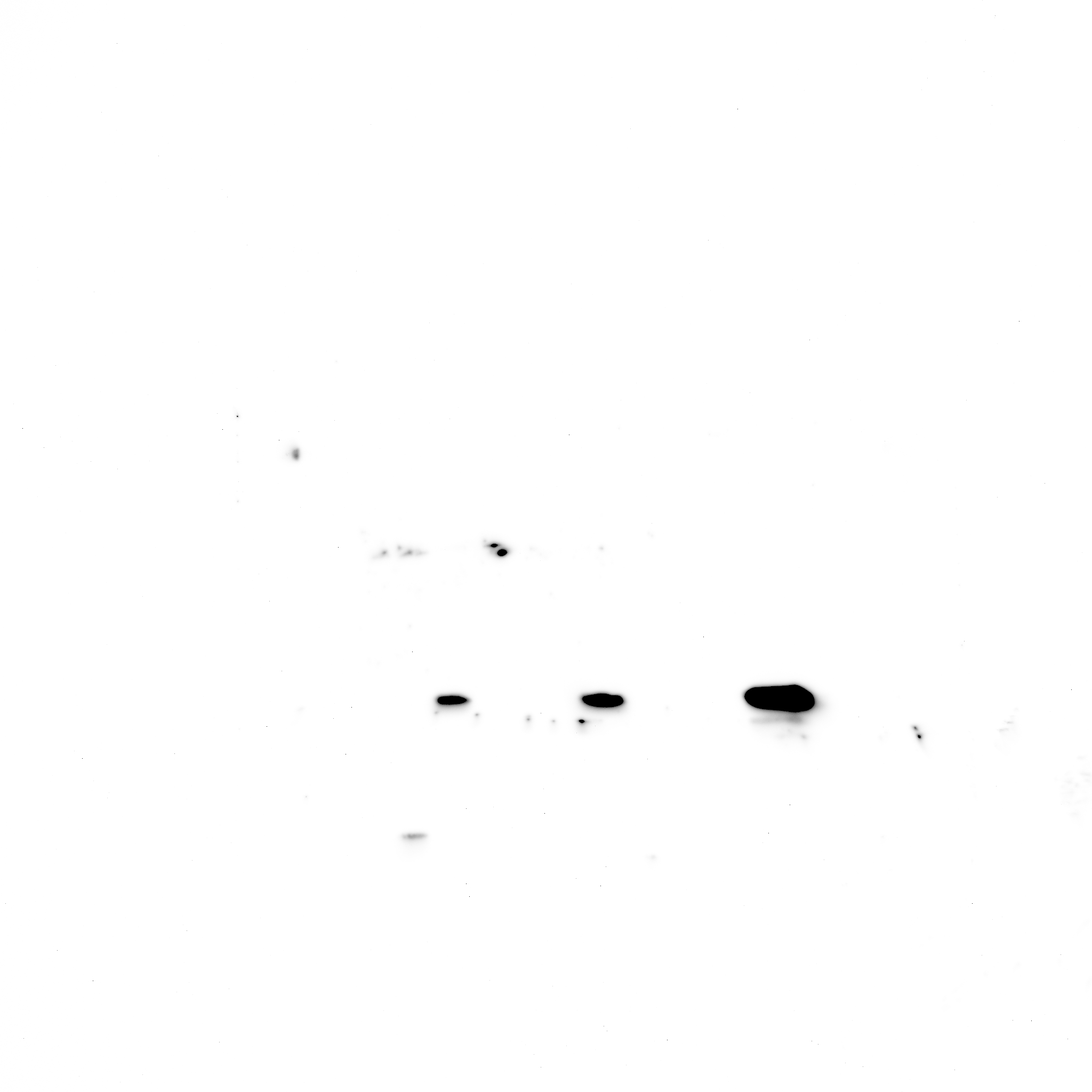

Supplement: Figure 1—source data 1. — Two close molecular markers are noted. FLAG-tagged TMEM263 at the predicted molecular weight is marked. The cropped region used for the main figure image is marked with a dotted box. This membrane was probed with the anti-FLAG antibody. Bottom left – The same membrane as shown above, but overexposed and imaged with the light channel to show the entire membrane outline. The anti-FLAG antibody is very clean and therefore the membrane boarder is hard to see otherwise. Top right – Original uncropped membrane from imager showing blue channel only as a black and white image. Two close molecular markers are noted. FLAG-tagged TMEM263 at the predicted molecular weight is marked. The cropped region used for the main figure image is marked with a dotted box. This membrane was probed with the anti-FLAG antibody. Bottom left – The same membrane as shown above, but overexposed and imaged with the light channel to show the entire membrane outline. The anti-TMEM263 antibody is fairly clean and therefore the membrane boarder is hard to see otherwise. [file elife-90949-fig1-data1.zip › Figure 1-source data 1/Figure 1-source data 1 - original uncropped - immunoblot of anti-TMEM263.tif]

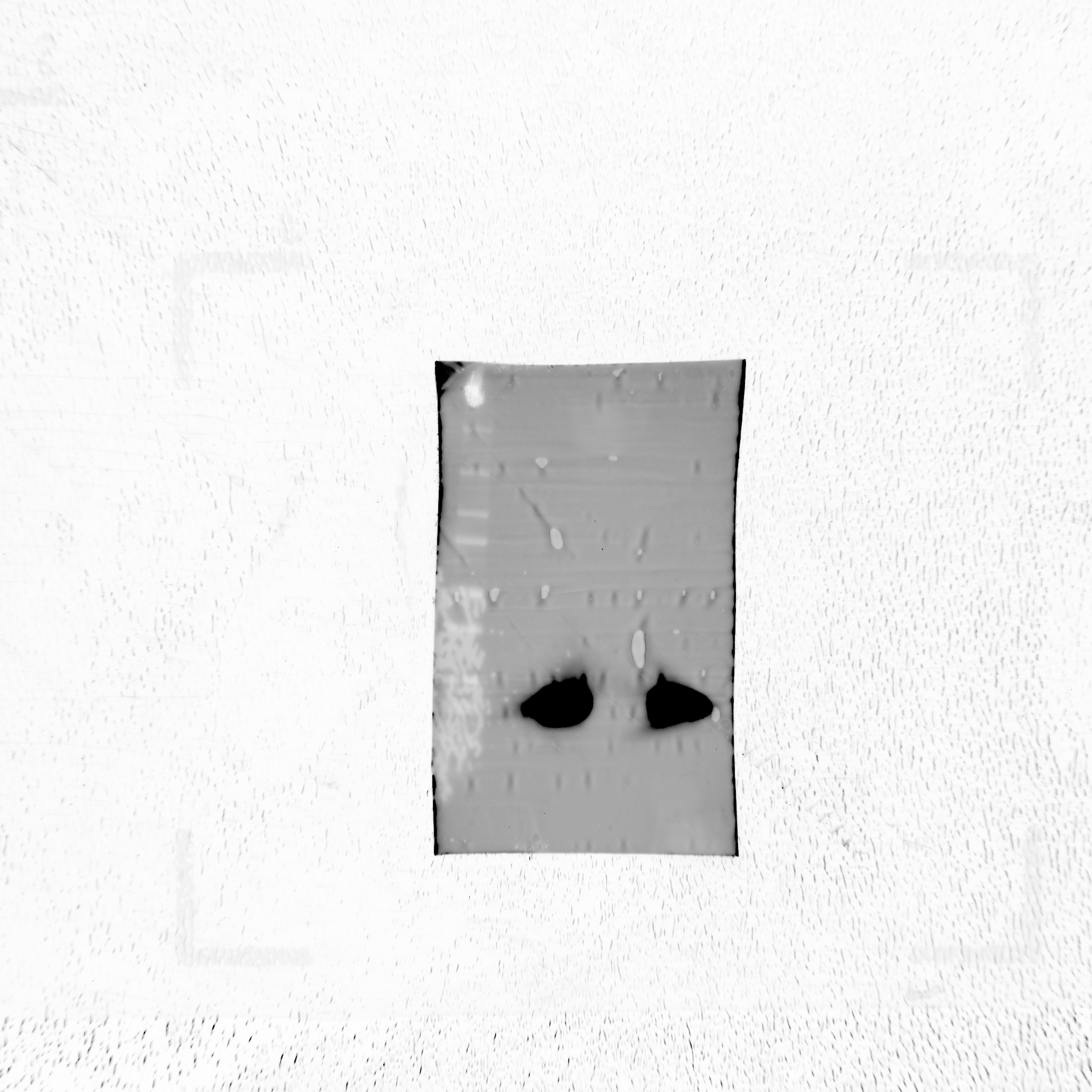

Supplement: Figure 1—source data 1. — Two close molecular markers are noted. FLAG-tagged TMEM263 at the predicted molecular weight is marked. The cropped region used for the main figure image is marked with a dotted box. This membrane was probed with the anti-FLAG antibody. Bottom left – The same membrane as shown above, but overexposed and imaged with the light channel to show the entire membrane outline. The anti-FLAG antibody is very clean and therefore the membrane boarder is hard to see otherwise. Top right – Original uncropped membrane from imager showing blue channel only as a black and white image. Two close molecular markers are noted. FLAG-tagged TMEM263 at the predicted molecular weight is marked. The cropped region used for the main figure image is marked with a dotted box. This membrane was probed with the anti-FLAG antibody. Bottom left – The same membrane as shown above, but overexposed and imaged with the light channel to show the entire membrane outline. The anti-TMEM263 antibody is fairly clean and therefore the membrane boarder is hard to see otherwise. [file elife-90949-fig1-data1.zip › Figure 1-source data 1/Figure 1-source data 1 - original uncropped - light image overlay on overexposed membrane - anti-FLAG.tif]

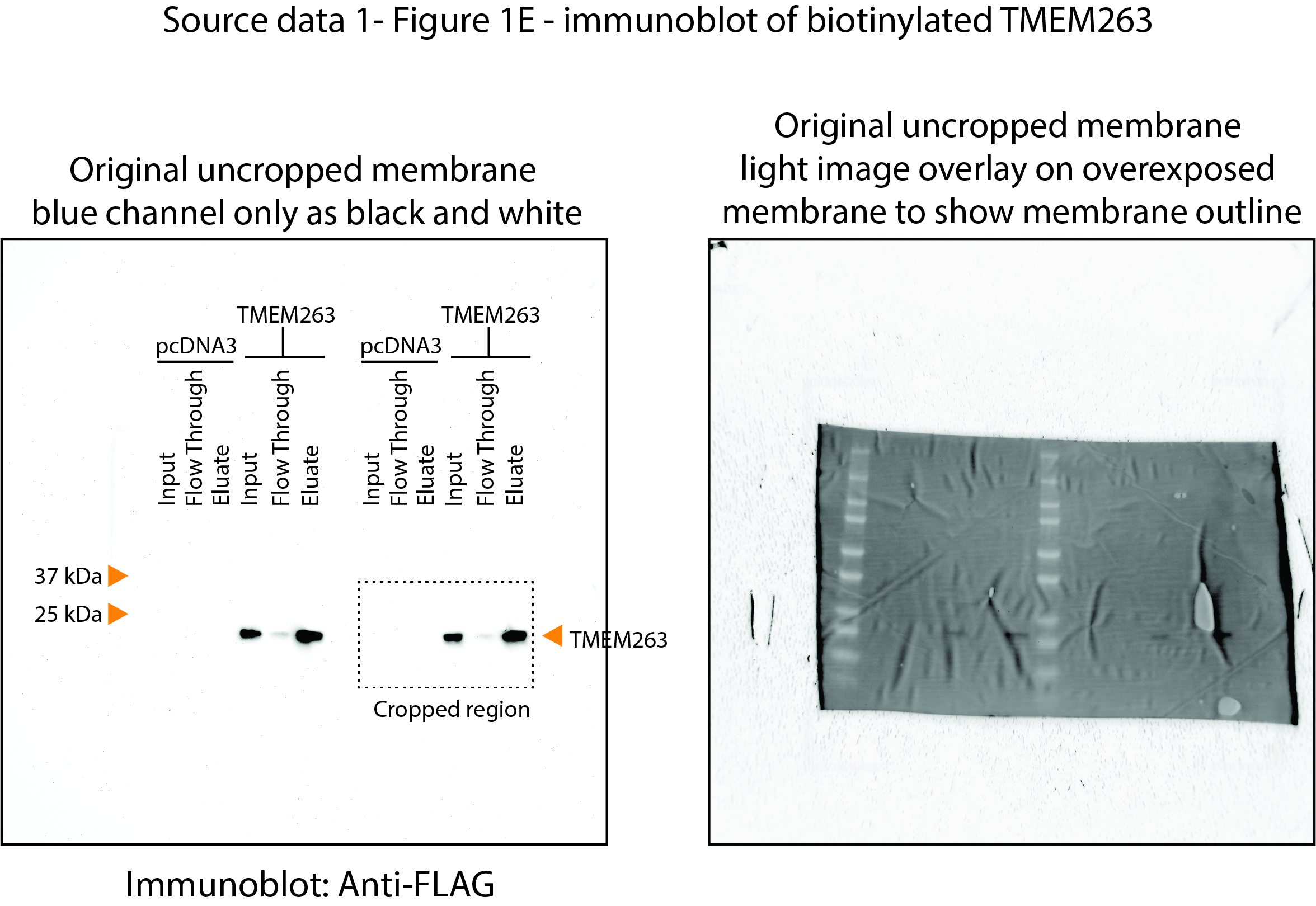

Supplement: Figure 1—source data 2. — Two close molecular markers are noted. FLAG-tagged TMEM263 at the predicted molecular weight is marked. The cropped region used for the main figure image is marked with a dotted box. This membrane was probed with the anti-FLAG antibody. Right – The same membrane as on the left, but overexposed and imaged with the light channel to show the entire membrane outline. The anti-FLAG antibody is very clean and therefore the membrane boarder is hard to see otherwise. [file elife-90949-fig1-data2.zip › Figure 1-source data 2/Figure 1-source data 2.tif]

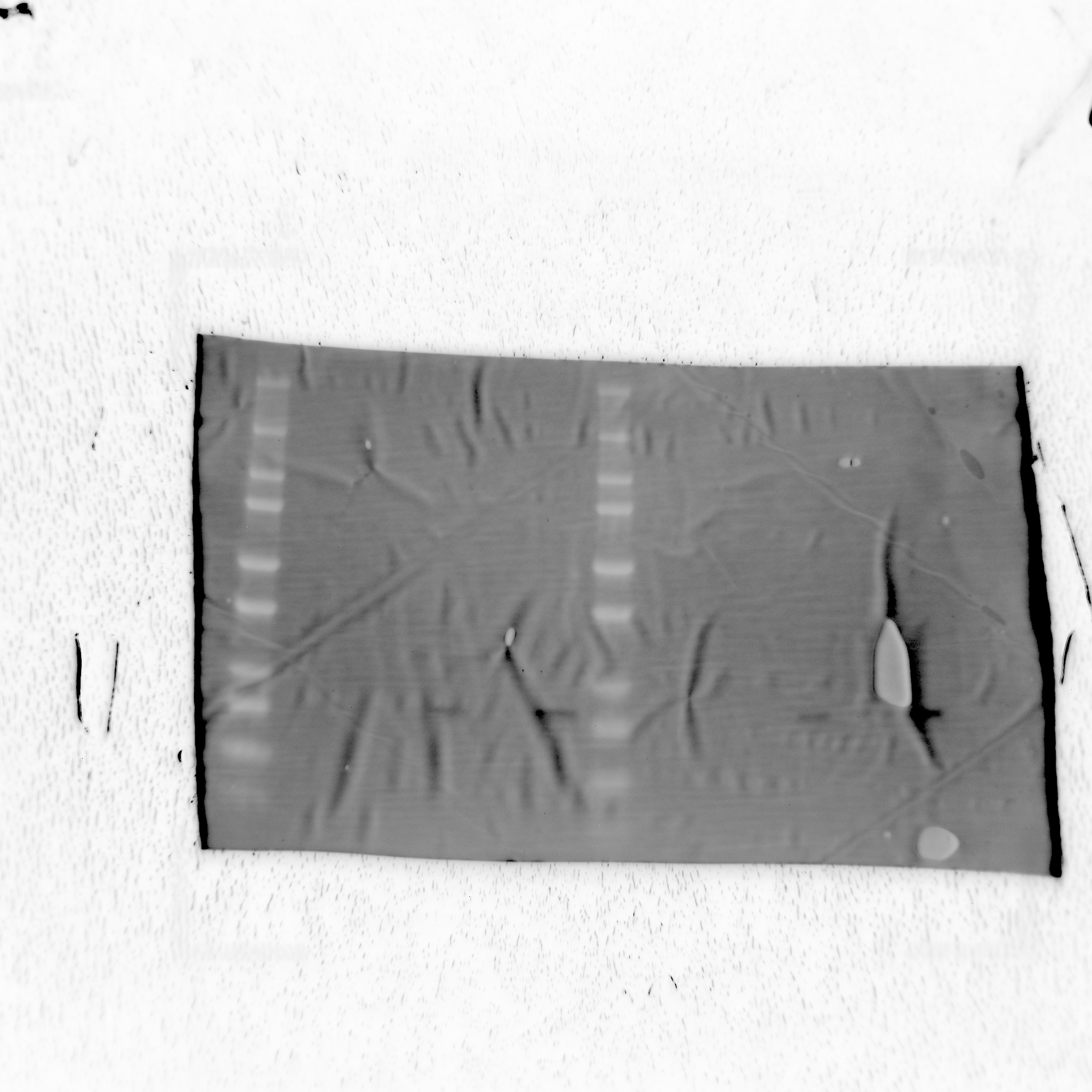

Supplement: Figure 1—source data 2. — Two close molecular markers are noted. FLAG-tagged TMEM263 at the predicted molecular weight is marked. The cropped region used for the main figure image is marked with a dotted box. This membrane was probed with the anti-FLAG antibody. Right – The same membrane as on the left, but overexposed and imaged with the light channel to show the entire membrane outline. The anti-FLAG antibody is very clean and therefore the membrane boarder is hard to see otherwise. [file elife-90949-fig1-data2.zip › Figure 1-source data 2/Figure 1-source data 2 - original uncropped - light image overlay on overexposed membrane - biotinylated TMEM263.tif]

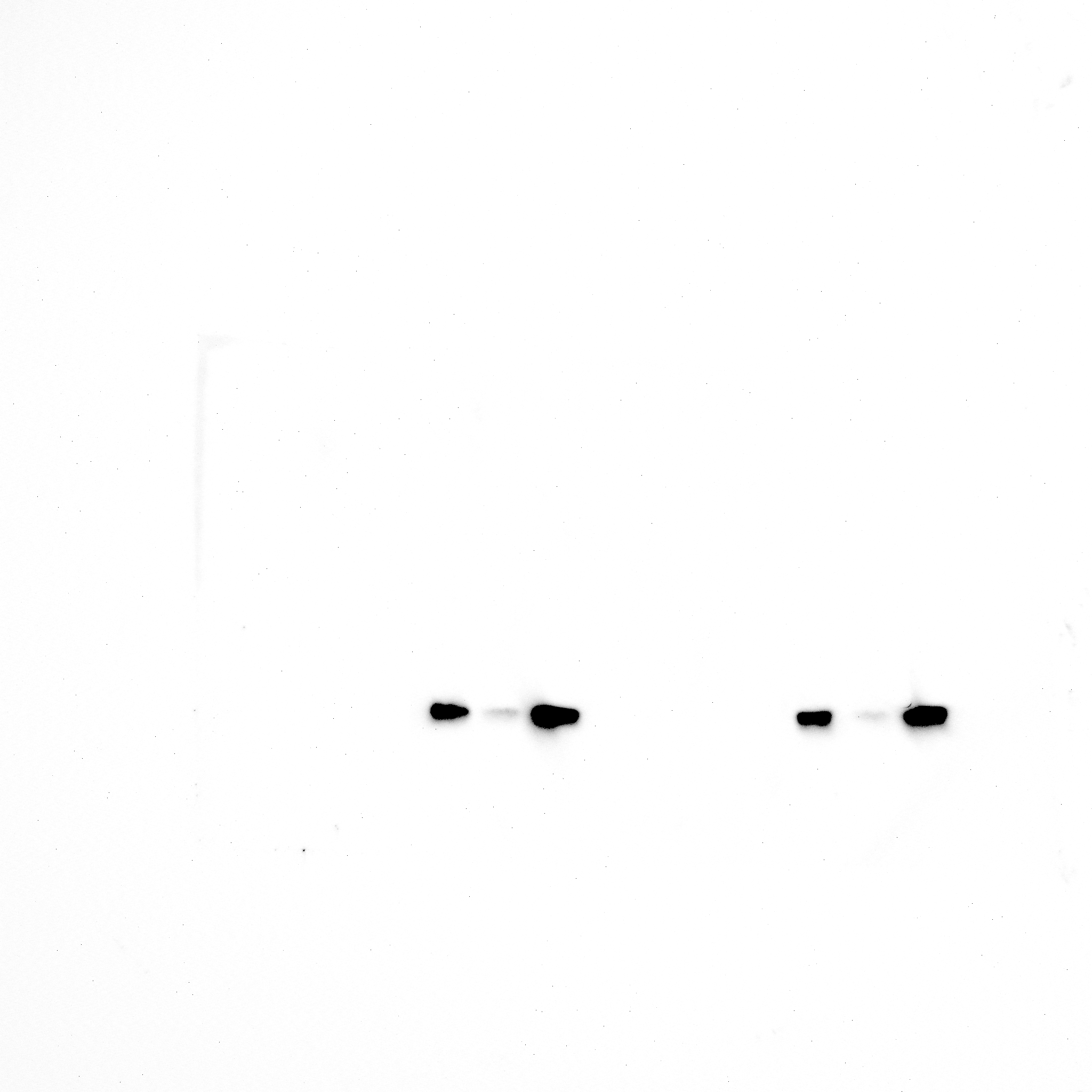

Supplement: Figure 1—source data 2. — Two close molecular markers are noted. FLAG-tagged TMEM263 at the predicted molecular weight is marked. The cropped region used for the main figure image is marked with a dotted box. This membrane was probed with the anti-FLAG antibody. Right – The same membrane as on the left, but overexposed and imaged with the light channel to show the entire membrane outline. The anti-FLAG antibody is very clean and therefore the membrane boarder is hard to see otherwise. [file elife-90949-fig1-data2.zip › Figure 1-source data 2/Figure 1-source data 2 - original uncropped - immunoblot of biotinylated TMEM263.tif]

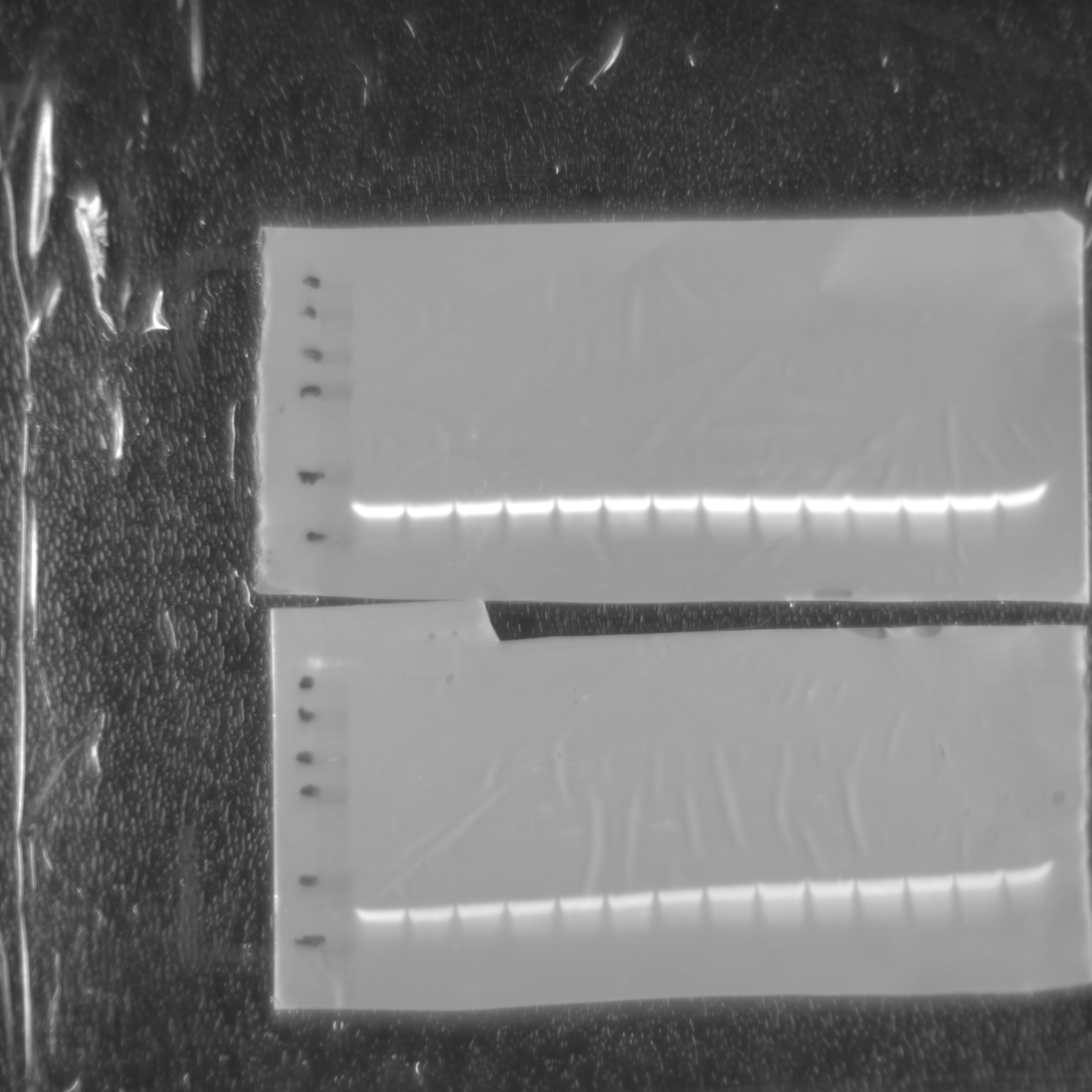

Supplement: Figure 5—source data 1. — Two close molecular markers are noted. Growth hormone receptor (GHR) at the predicted molecular weight is marked. The cropped region used for the main figure image is marked with a dotted box. This membrane was probed with the anti-GHR antibody. Top middle – Original uncropped membrane from imager showing blue channel only as black and white. Two molecular markers are noted. β-Actin at the predicted molecular weight is marked. The cropped region used for the main figure is marked with a dotted box. The membrane was probed with the anti-β-actin antibody. Top right – The same membrane as the top middle, but overexposed and imaged with the light channel to show the entire membrane outline. The anti-β-actin antibody is very clean and therefore the membrane boarder is hard to see otherwise. Bottom left – Original uncropped membrane from imager showing blue channel only as a black and white image. Two close molecular markers are noted. GHR at the predicted molecular weight is marked. The cropped region used for the main figure image is marked with a dotted box. This membrane was probed with the anti-GHR antibody. Bottom middle – Original uncropped membrane from imager showing blue channel only as black and white. Two molecular markers are noted. β-Actin at the predicted molecular weight is marked. The cropped region used for the main figure is marked with a dotted box. The membrane was probed with the anti-β-actin antibody. Bottom right – The same membrane as the top middle, but overexposed and imaged with the light channel to show the entire membrane outline. The anti-β-actin antibody is very clean and therefore the membrane boarder is hard to see otherwise. [file elife-90949-fig5-data1.zip › Figure 5-source data 1/Figure 5-source data 1 - original uncropped - light image overlay of overexposed membrane - male Anti-B-Actin.tif]

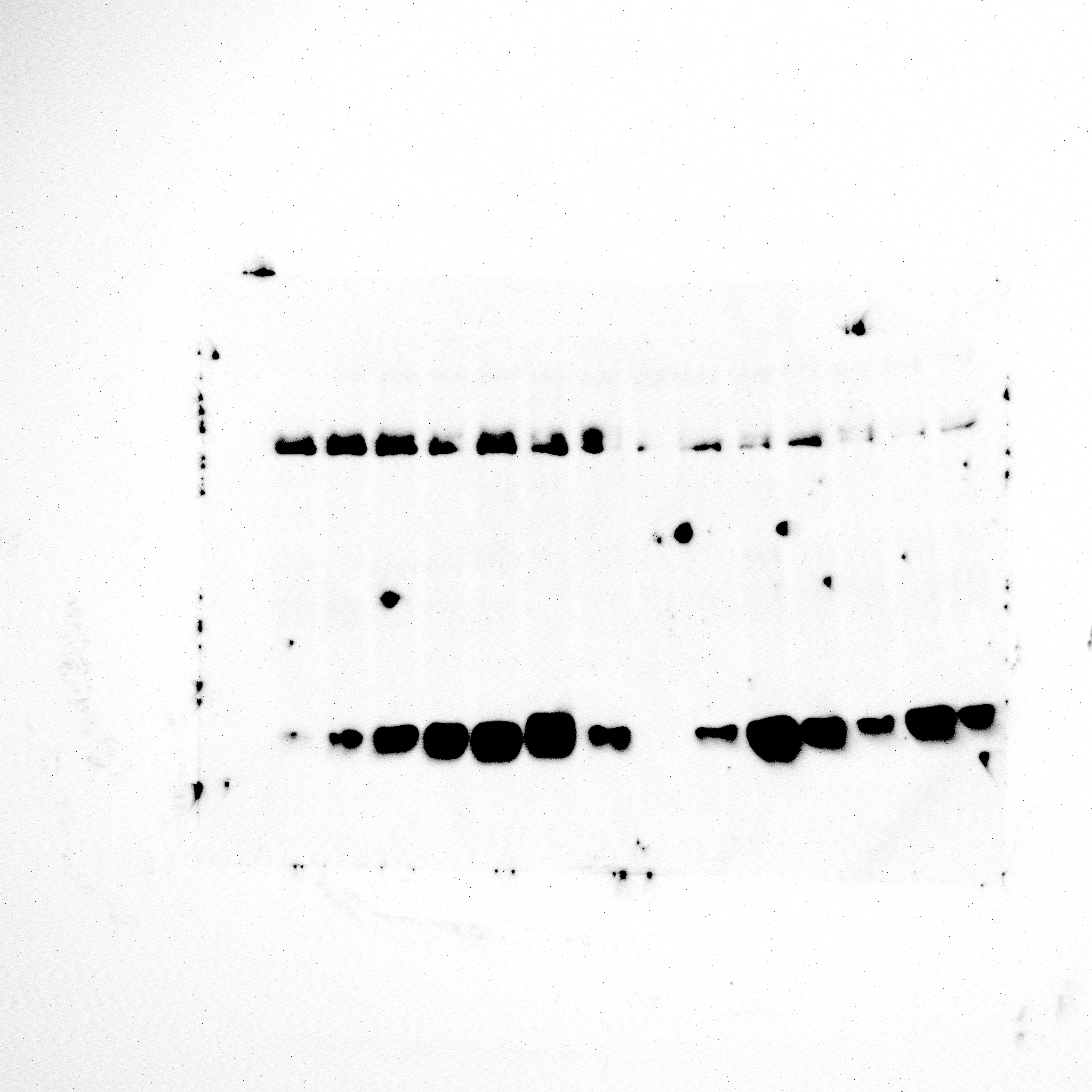

Supplement: Figure 5—source data 1. — Two close molecular markers are noted. Growth hormone receptor (GHR) at the predicted molecular weight is marked. The cropped region used for the main figure image is marked with a dotted box. This membrane was probed with the anti-GHR antibody. Top middle – Original uncropped membrane from imager showing blue channel only as black and white. Two molecular markers are noted. β-Actin at the predicted molecular weight is marked. The cropped region used for the main figure is marked with a dotted box. The membrane was probed with the anti-β-actin antibody. Top right – The same membrane as the top middle, but overexposed and imaged with the light channel to show the entire membrane outline. The anti-β-actin antibody is very clean and therefore the membrane boarder is hard to see otherwise. Bottom left – Original uncropped membrane from imager showing blue channel only as a black and white image. Two close molecular markers are noted. GHR at the predicted molecular weight is marked. The cropped region used for the main figure image is marked with a dotted box. This membrane was probed with the anti-GHR antibody. Bottom middle – Original uncropped membrane from imager showing blue channel only as black and white. Two molecular markers are noted. β-Actin at the predicted molecular weight is marked. The cropped region used for the main figure is marked with a dotted box. The membrane was probed with the anti-β-actin antibody. Bottom right – The same membrane as the top middle, but overexposed and imaged with the light channel to show the entire membrane outline. The anti-β-actin antibody is very clean and therefore the membrane boarder is hard to see otherwise. [file elife-90949-fig5-data1.zip › Figure 5-source data 1/Figure 5-source data 1 - original uncropped - immunoblot of female GH-R.tif]

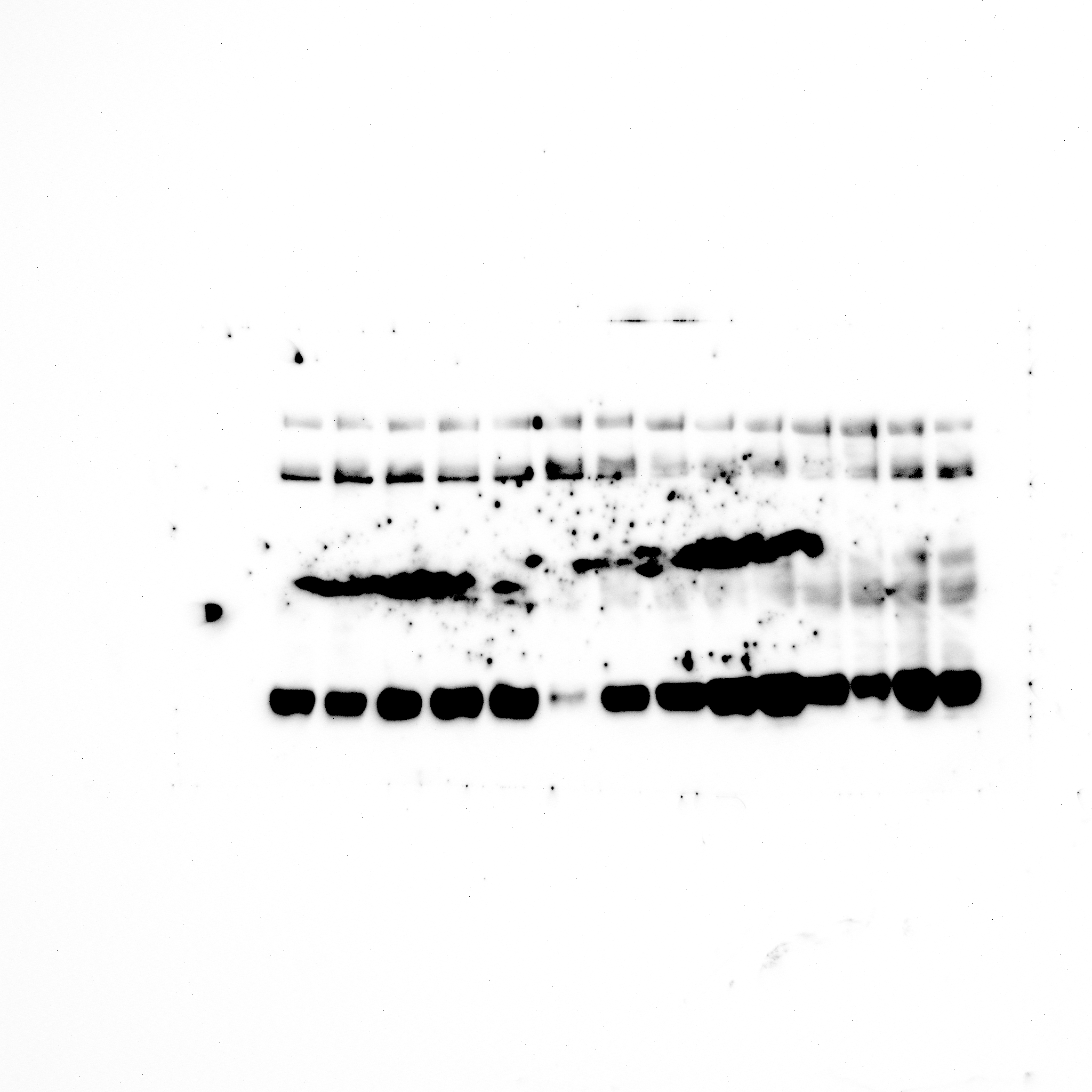

Supplement: Figure 5—source data 1. — Two close molecular markers are noted. Growth hormone receptor (GHR) at the predicted molecular weight is marked. The cropped region used for the main figure image is marked with a dotted box. This membrane was probed with the anti-GHR antibody. Top middle – Original uncropped membrane from imager showing blue channel only as black and white. Two molecular markers are noted. β-Actin at the predicted molecular weight is marked. The cropped region used for the main figure is marked with a dotted box. The membrane was probed with the anti-β-actin antibody. Top right – The same membrane as the top middle, but overexposed and imaged with the light channel to show the entire membrane outline. The anti-β-actin antibody is very clean and therefore the membrane boarder is hard to see otherwise. Bottom left – Original uncropped membrane from imager showing blue channel only as a black and white image. Two close molecular markers are noted. GHR at the predicted molecular weight is marked. The cropped region used for the main figure image is marked with a dotted box. This membrane was probed with the anti-GHR antibody. Bottom middle – Original uncropped membrane from imager showing blue channel only as black and white. Two molecular markers are noted. β-Actin at the predicted molecular weight is marked. The cropped region used for the main figure is marked with a dotted box. The membrane was probed with the anti-β-actin antibody. Bottom right – The same membrane as the top middle, but overexposed and imaged with the light channel to show the entire membrane outline. The anti-β-actin antibody is very clean and therefore the membrane boarder is hard to see otherwise. [file elife-90949-fig5-data1.zip › Figure 5-source data 1/Figure 5-source data 1 - original uncropped - immunoblot of male GH-R.tif]

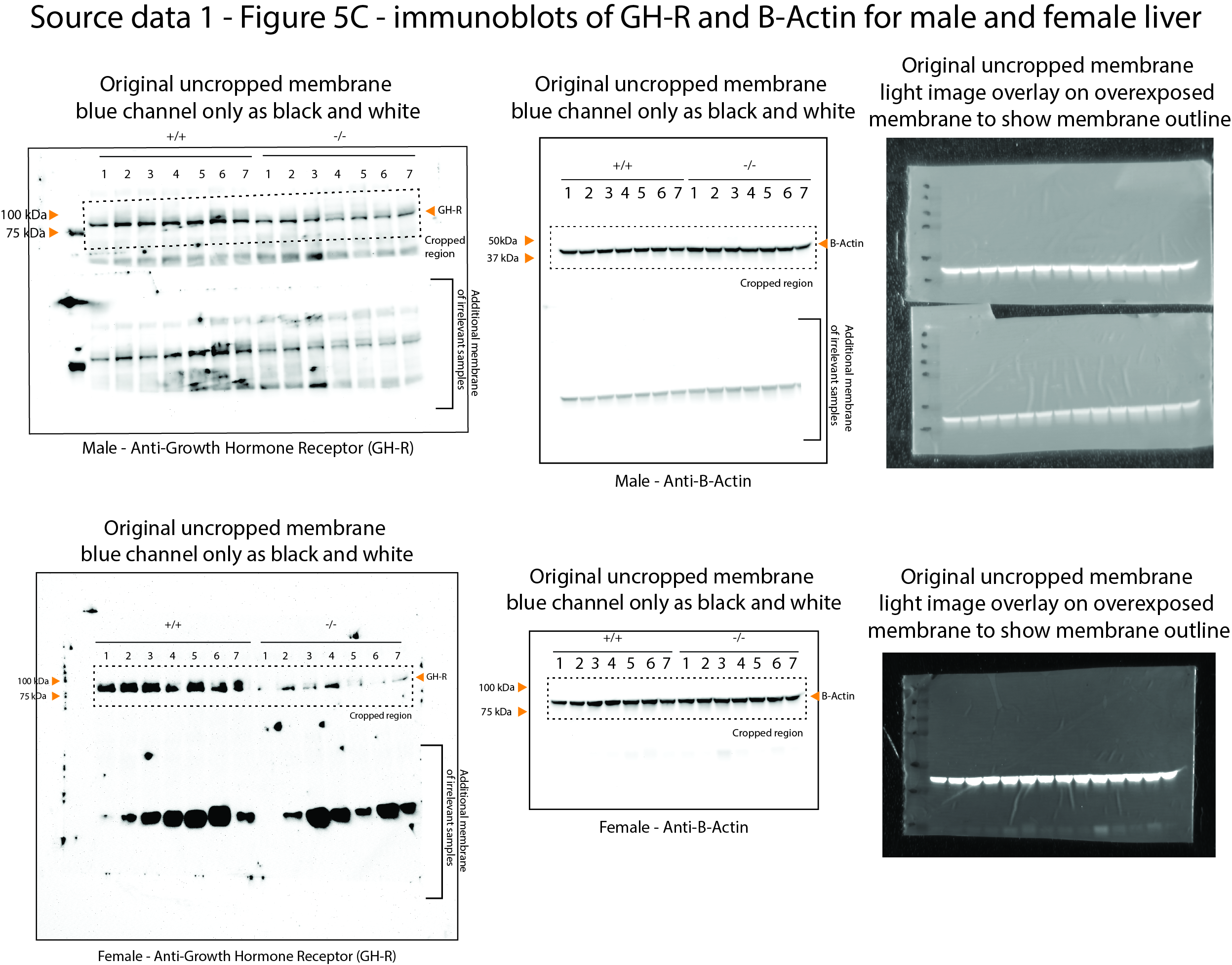

Supplement: Figure 5—source data 1. — Two close molecular markers are noted. Growth hormone receptor (GHR) at the predicted molecular weight is marked. The cropped region used for the main figure image is marked with a dotted box. This membrane was probed with the anti-GHR antibody. Top middle – Original uncropped membrane from imager showing blue channel only as black and white. Two molecular markers are noted. β-Actin at the predicted molecular weight is marked. The cropped region used for the main figure is marked with a dotted box. The membrane was probed with the anti-β-actin antibody. Top right – The same membrane as the top middle, but overexposed and imaged with the light channel to show the entire membrane outline. The anti-β-actin antibody is very clean and therefore the membrane boarder is hard to see otherwise. Bottom left – Original uncropped membrane from imager showing blue channel only as a black and white image. Two close molecular markers are noted. GHR at the predicted molecular weight is marked. The cropped region used for the main figure image is marked with a dotted box. This membrane was probed with the anti-GHR antibody. Bottom middle – Original uncropped membrane from imager showing blue channel only as black and white. Two molecular markers are noted. β-Actin at the predicted molecular weight is marked. The cropped region used for the main figure is marked with a dotted box. The membrane was probed with the anti-β-actin antibody. Bottom right – The same membrane as the top middle, but overexposed and imaged with the light channel to show the entire membrane outline. The anti-β-actin antibody is very clean and therefore the membrane boarder is hard to see otherwise. [file elife-90949-fig5-data1.zip › Figure 5-source data 1/Figure 5-source data 1.tif]

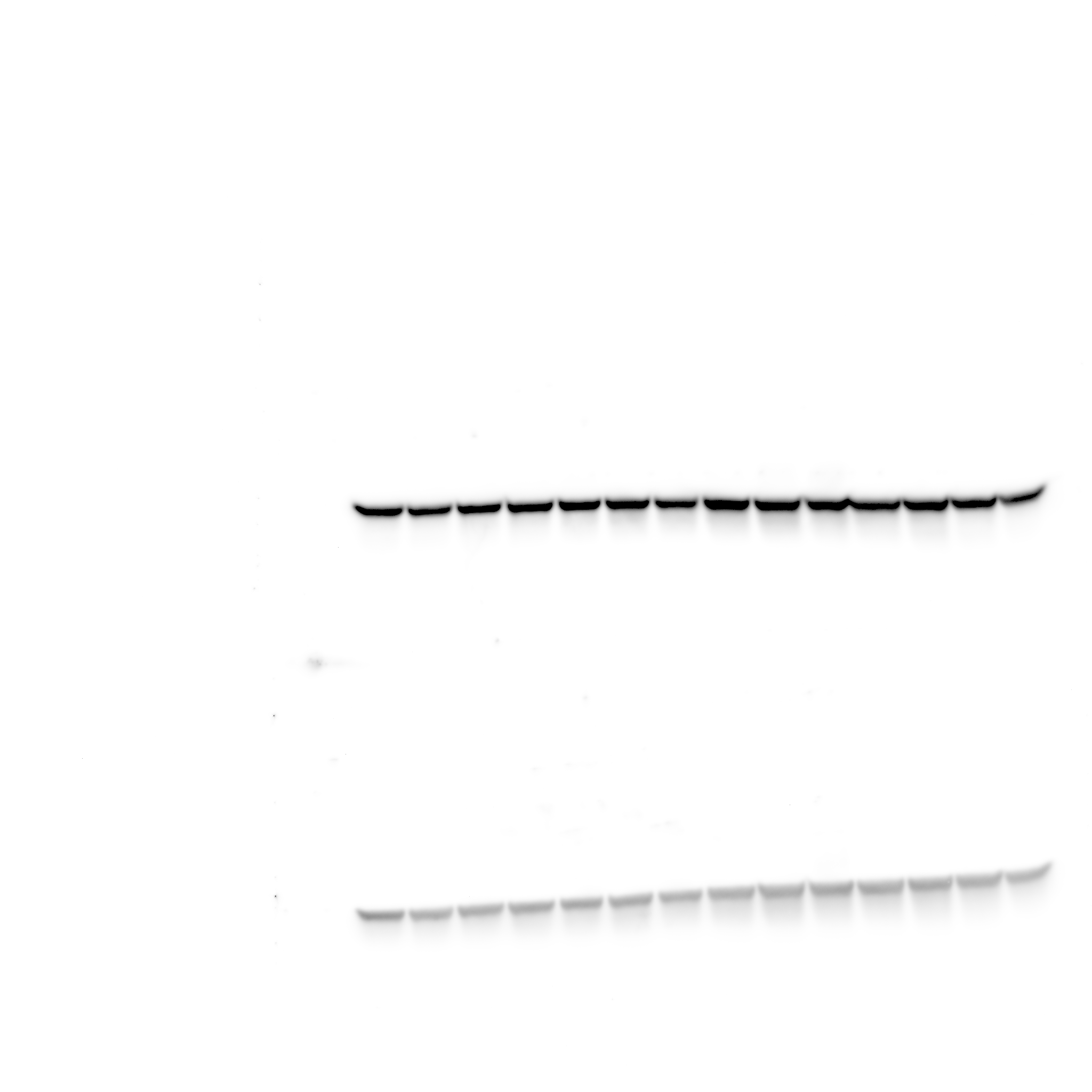

Supplement: Figure 5—source data 1. — Two close molecular markers are noted. Growth hormone receptor (GHR) at the predicted molecular weight is marked. The cropped region used for the main figure image is marked with a dotted box. This membrane was probed with the anti-GHR antibody. Top middle – Original uncropped membrane from imager showing blue channel only as black and white. Two molecular markers are noted. β-Actin at the predicted molecular weight is marked. The cropped region used for the main figure is marked with a dotted box. The membrane was probed with the anti-β-actin antibody. Top right – The same membrane as the top middle, but overexposed and imaged with the light channel to show the entire membrane outline. The anti-β-actin antibody is very clean and therefore the membrane boarder is hard to see otherwise. Bottom left – Original uncropped membrane from imager showing blue channel only as a black and white image. Two close molecular markers are noted. GHR at the predicted molecular weight is marked. The cropped region used for the main figure image is marked with a dotted box. This membrane was probed with the anti-GHR antibody. Bottom middle – Original uncropped membrane from imager showing blue channel only as black and white. Two molecular markers are noted. β-Actin at the predicted molecular weight is marked. The cropped region used for the main figure is marked with a dotted box. The membrane was probed with the anti-β-actin antibody. Bottom right – The same membrane as the top middle, but overexposed and imaged with the light channel to show the entire membrane outline. The anti-β-actin antibody is very clean and therefore the membrane boarder is hard to see otherwise. [file elife-90949-fig5-data1.zip › Figure 5-source data 1/Figure 5-source data 1 - original uncropped - immunoblot of male B-Actin.tif]

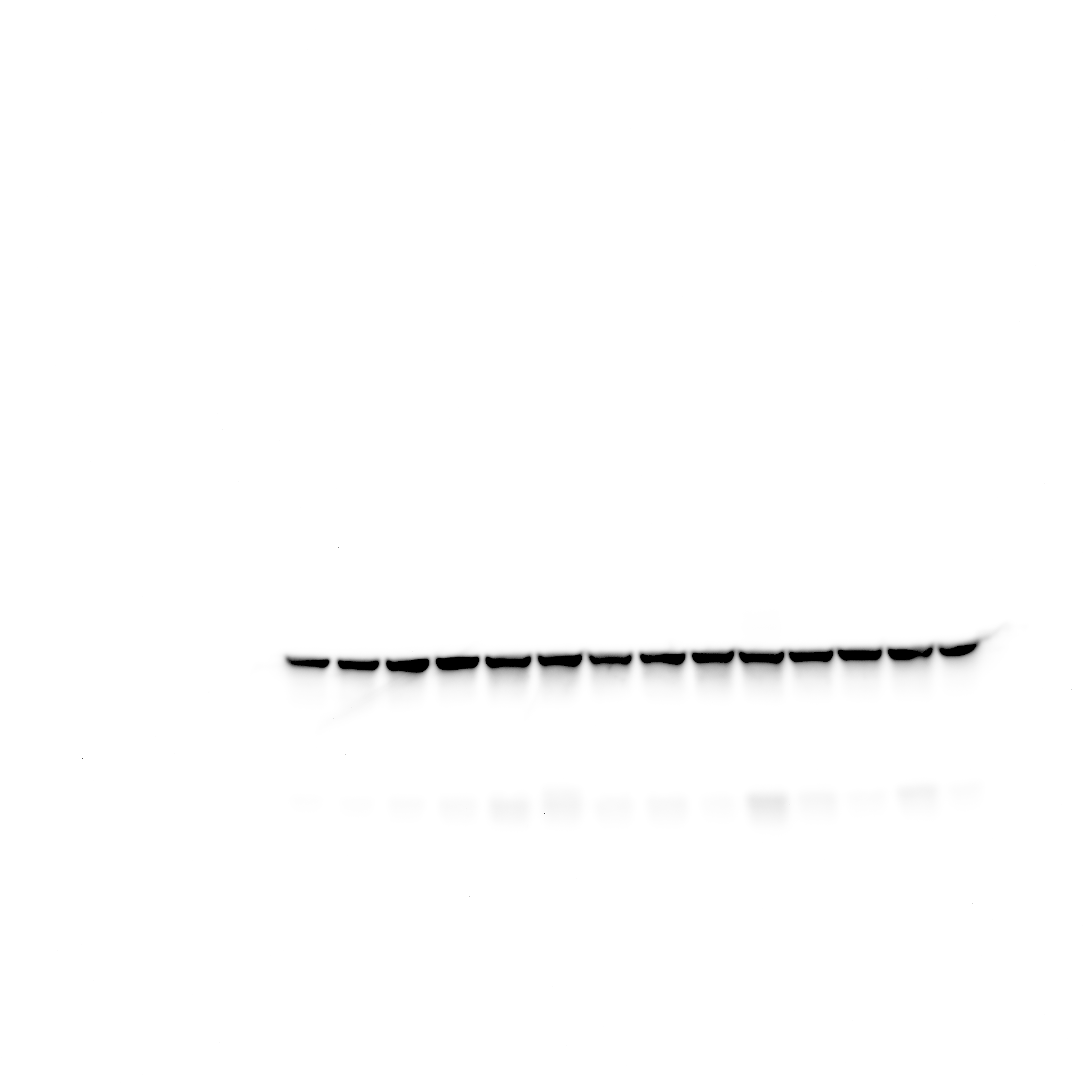

Supplement: Figure 5—source data 1. — Two close molecular markers are noted. Growth hormone receptor (GHR) at the predicted molecular weight is marked. The cropped region used for the main figure image is marked with a dotted box. This membrane was probed with the anti-GHR antibody. Top middle – Original uncropped membrane from imager showing blue channel only as black and white. Two molecular markers are noted. β-Actin at the predicted molecular weight is marked. The cropped region used for the main figure is marked with a dotted box. The membrane was probed with the anti-β-actin antibody. Top right – The same membrane as the top middle, but overexposed and imaged with the light channel to show the entire membrane outline. The anti-β-actin antibody is very clean and therefore the membrane boarder is hard to see otherwise. Bottom left – Original uncropped membrane from imager showing blue channel only as a black and white image. Two close molecular markers are noted. GHR at the predicted molecular weight is marked. The cropped region used for the main figure image is marked with a dotted box. This membrane was probed with the anti-GHR antibody. Bottom middle – Original uncropped membrane from imager showing blue channel only as black and white. Two molecular markers are noted. β-Actin at the predicted molecular weight is marked. The cropped region used for the main figure is marked with a dotted box. The membrane was probed with the anti-β-actin antibody. Bottom right – The same membrane as the top middle, but overexposed and imaged with the light channel to show the entire membrane outline. The anti-β-actin antibody is very clean and therefore the membrane boarder is hard to see otherwise. [file elife-90949-fig5-data1.zip › Figure 5-source data 1/Figure 5-source data 1 - original uncropped - immunoblot of female B-Actin.tif]

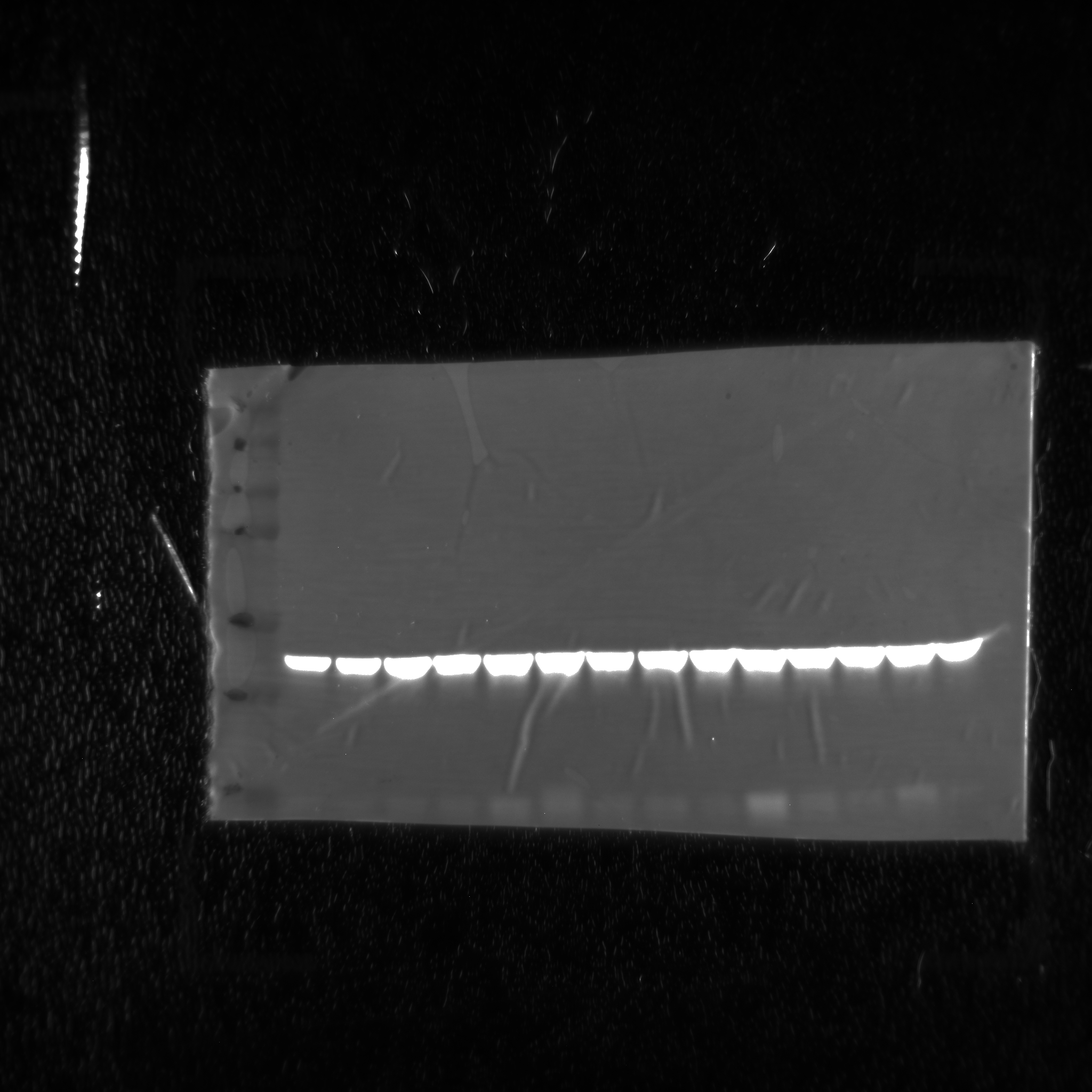

Supplement: Figure 5—source data 1. — Two close molecular markers are noted. Growth hormone receptor (GHR) at the predicted molecular weight is marked. The cropped region used for the main figure image is marked with a dotted box. This membrane was probed with the anti-GHR antibody. Top middle – Original uncropped membrane from imager showing blue channel only as black and white. Two molecular markers are noted. β-Actin at the predicted molecular weight is marked. The cropped region used for the main figure is marked with a dotted box. The membrane was probed with the anti-β-actin antibody. Top right – The same membrane as the top middle, but overexposed and imaged with the light channel to show the entire membrane outline. The anti-β-actin antibody is very clean and therefore the membrane boarder is hard to see otherwise. Bottom left – Original uncropped membrane from imager showing blue channel only as a black and white image. Two close molecular markers are noted. GHR at the predicted molecular weight is marked. The cropped region used for the main figure image is marked with a dotted box. This membrane was probed with the anti-GHR antibody. Bottom middle – Original uncropped membrane from imager showing blue channel only as black and white. Two molecular markers are noted. β-Actin at the predicted molecular weight is marked. The cropped region used for the main figure is marked with a dotted box. The membrane was probed with the anti-β-actin antibody. Bottom right – The same membrane as the top middle, but overexposed and imaged with the light channel to show the entire membrane outline. The anti-β-actin antibody is very clean and therefore the membrane boarder is hard to see otherwise. [file elife-90949-fig5-data1.zip › Figure 5-source data 1/Figure 5-source data 1 - original uncropped - light image overlay of overexposed membrane - female Anti-B-Actin.tif]

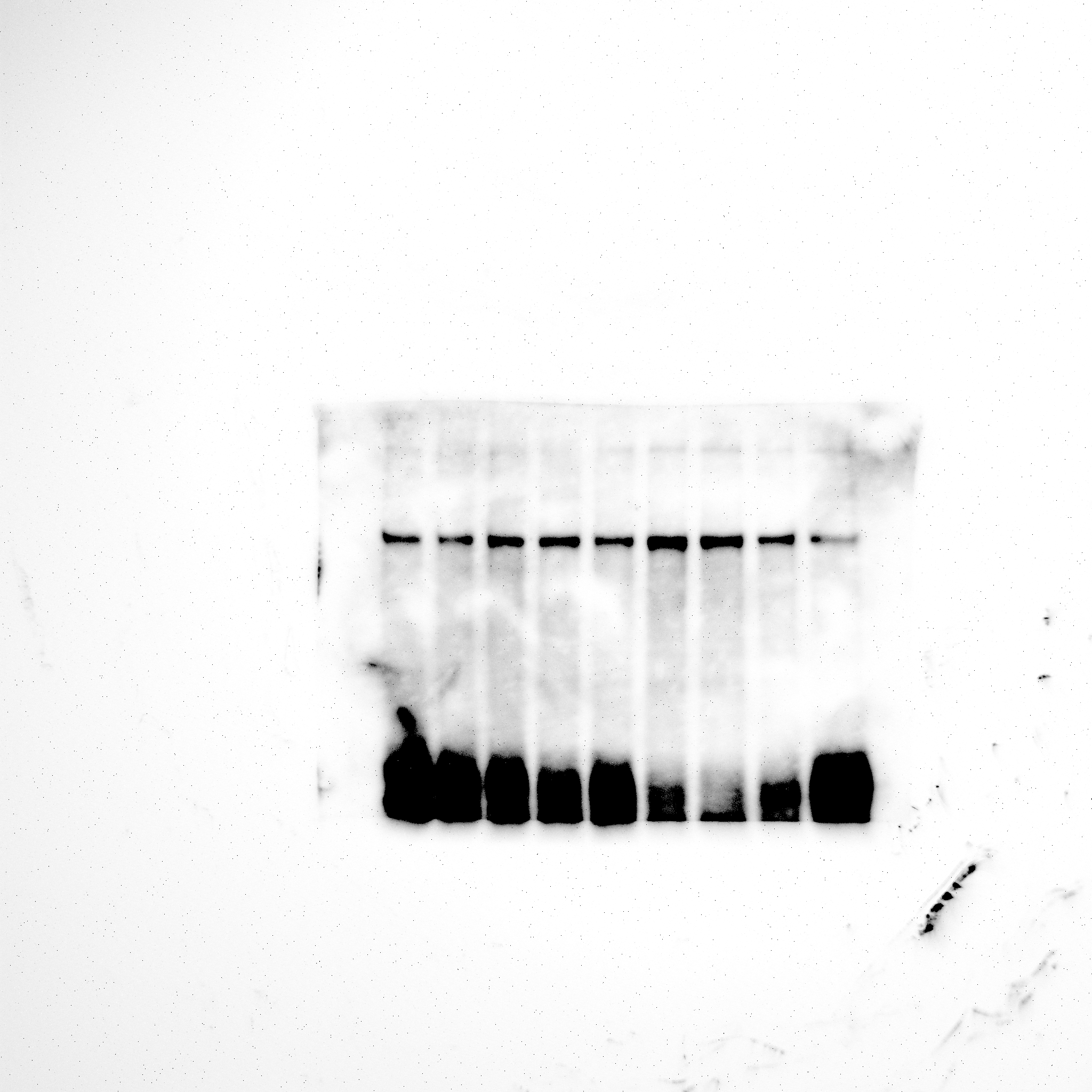

Supplement: Figure 5—source data 2. — Two close molecular markers are noted. Phospho-JAK2 at the predicted molecular weight is marked. The cropped region used for the main figure image is marked with a dotted box. This membrane was probed with the anti-phospho-JAK2 antibody. Top right – The same membrane as on the left, re-probed to visualize total-JAK2 signal. Bottom left – Original uncropped membrane from imager showing blue channel only as a black and white image. Two close molecular markers are noted. Phospho-STAT5b at the predicted molecular weight is marked. The cropped region used for the main figure image is marked with a dotted box. This membrane was probed with the anti-phospho-STAT5b antibody. Bottom right – The same membrane as on the bottom left, re-probed to visualize total-STAT5b signal. [file elife-90949-fig5-data2.zip › Figure 5-source data 2/Figure 5-source data 2 - original uncropped - immunoblot of male total-JAK2 after GH injection.tif]

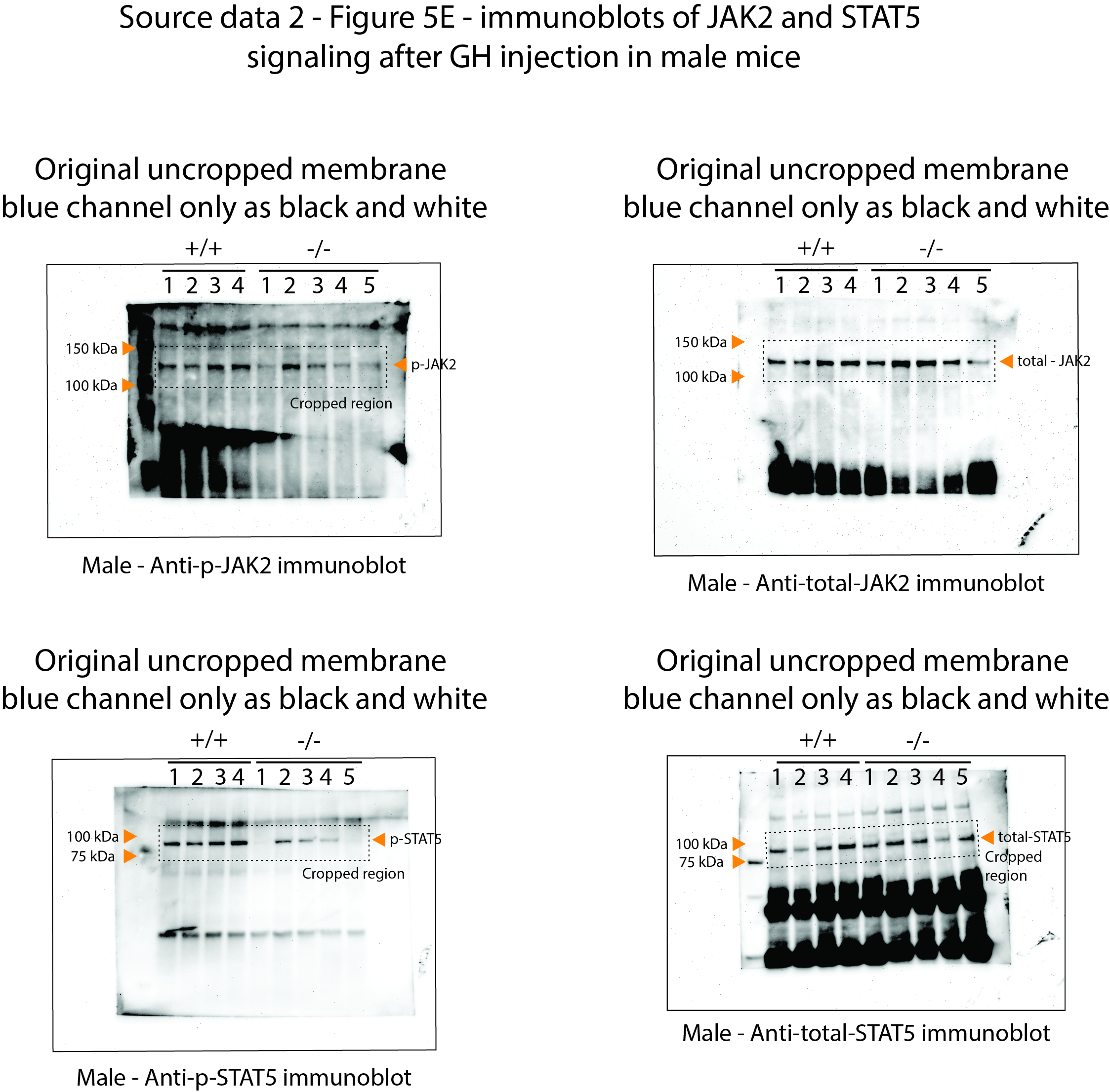

Supplement: Figure 5—source data 2. — Two close molecular markers are noted. Phospho-JAK2 at the predicted molecular weight is marked. The cropped region used for the main figure image is marked with a dotted box. This membrane was probed with the anti-phospho-JAK2 antibody. Top right – The same membrane as on the left, re-probed to visualize total-JAK2 signal. Bottom left – Original uncropped membrane from imager showing blue channel only as a black and white image. Two close molecular markers are noted. Phospho-STAT5b at the predicted molecular weight is marked. The cropped region used for the main figure image is marked with a dotted box. This membrane was probed with the anti-phospho-STAT5b antibody. Bottom right – The same membrane as on the bottom left, re-probed to visualize total-STAT5b signal. [file elife-90949-fig5-data2.zip › Figure 5-source data 2/Figure 5-source data 2.tif]

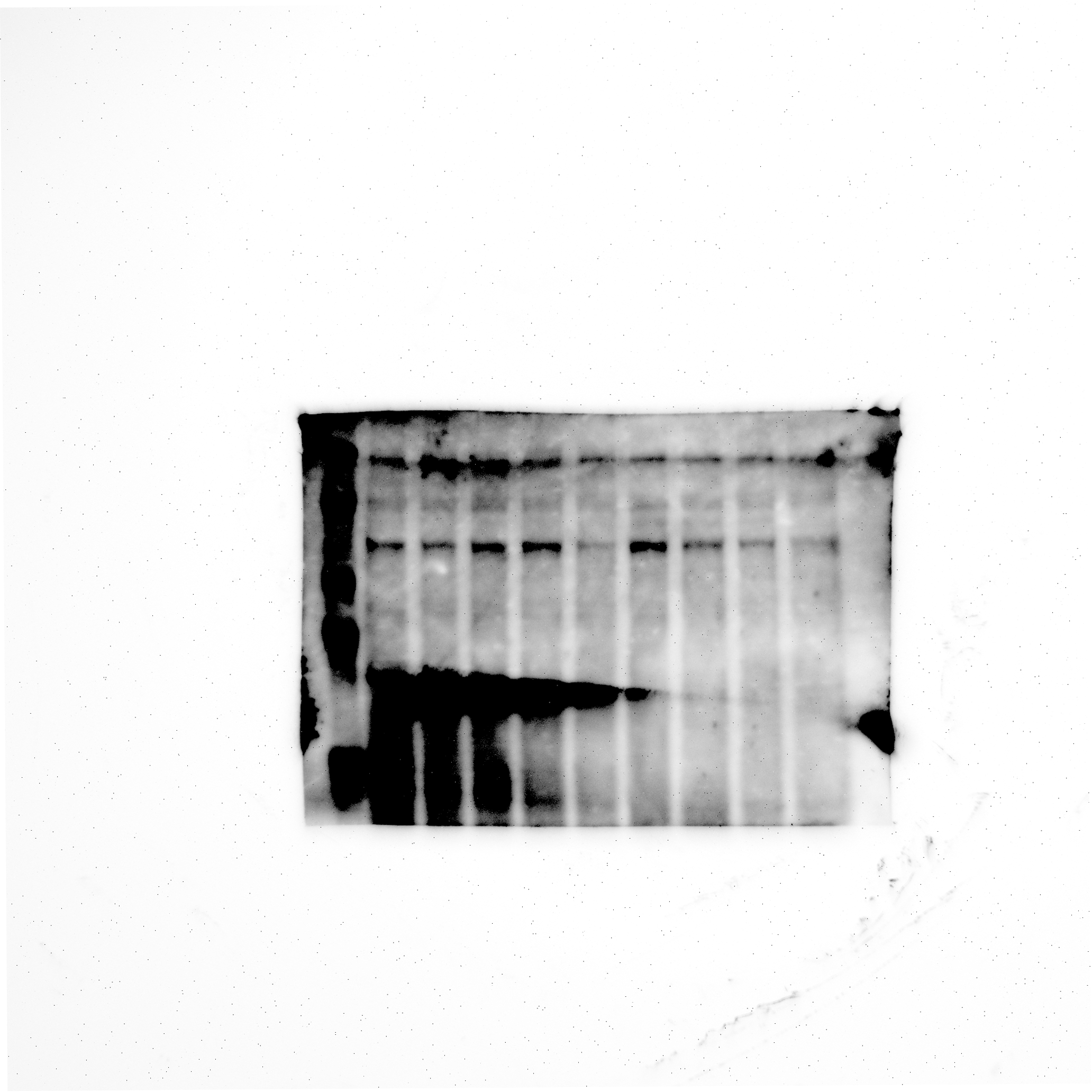

Supplement: Figure 5—source data 2. — Two close molecular markers are noted. Phospho-JAK2 at the predicted molecular weight is marked. The cropped region used for the main figure image is marked with a dotted box. This membrane was probed with the anti-phospho-JAK2 antibody. Top right – The same membrane as on the left, re-probed to visualize total-JAK2 signal. Bottom left – Original uncropped membrane from imager showing blue channel only as a black and white image. Two close molecular markers are noted. Phospho-STAT5b at the predicted molecular weight is marked. The cropped region used for the main figure image is marked with a dotted box. This membrane was probed with the anti-phospho-STAT5b antibody. Bottom right – The same membrane as on the bottom left, re-probed to visualize total-STAT5b signal. [file elife-90949-fig5-data2.zip › Figure 5-source data 2/Figure 5-source data 2 - original uncropped - immunoblot of male p-JAK2 after GH injection.tif]

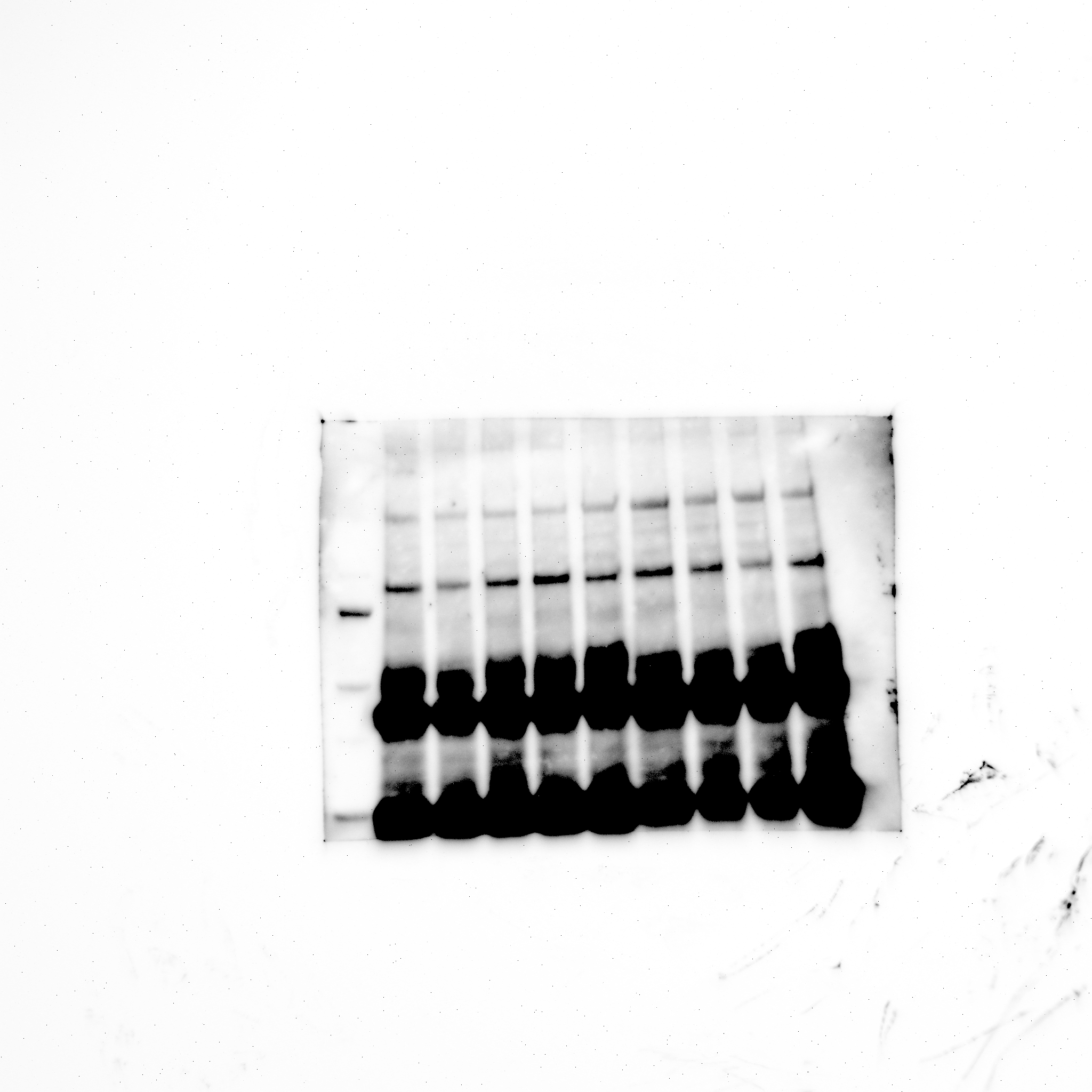

Supplement: Figure 5—source data 2. — Two close molecular markers are noted. Phospho-JAK2 at the predicted molecular weight is marked. The cropped region used for the main figure image is marked with a dotted box. This membrane was probed with the anti-phospho-JAK2 antibody. Top right – The same membrane as on the left, re-probed to visualize total-JAK2 signal. Bottom left – Original uncropped membrane from imager showing blue channel only as a black and white image. Two close molecular markers are noted. Phospho-STAT5b at the predicted molecular weight is marked. The cropped region used for the main figure image is marked with a dotted box. This membrane was probed with the anti-phospho-STAT5b antibody. Bottom right – The same membrane as on the bottom left, re-probed to visualize total-STAT5b signal. [file elife-90949-fig5-data2.zip › Figure 5-source data 2/Figure 5-source data 2 - original uncropped - immunoblot of total-STAT5 after GH injection.tif]

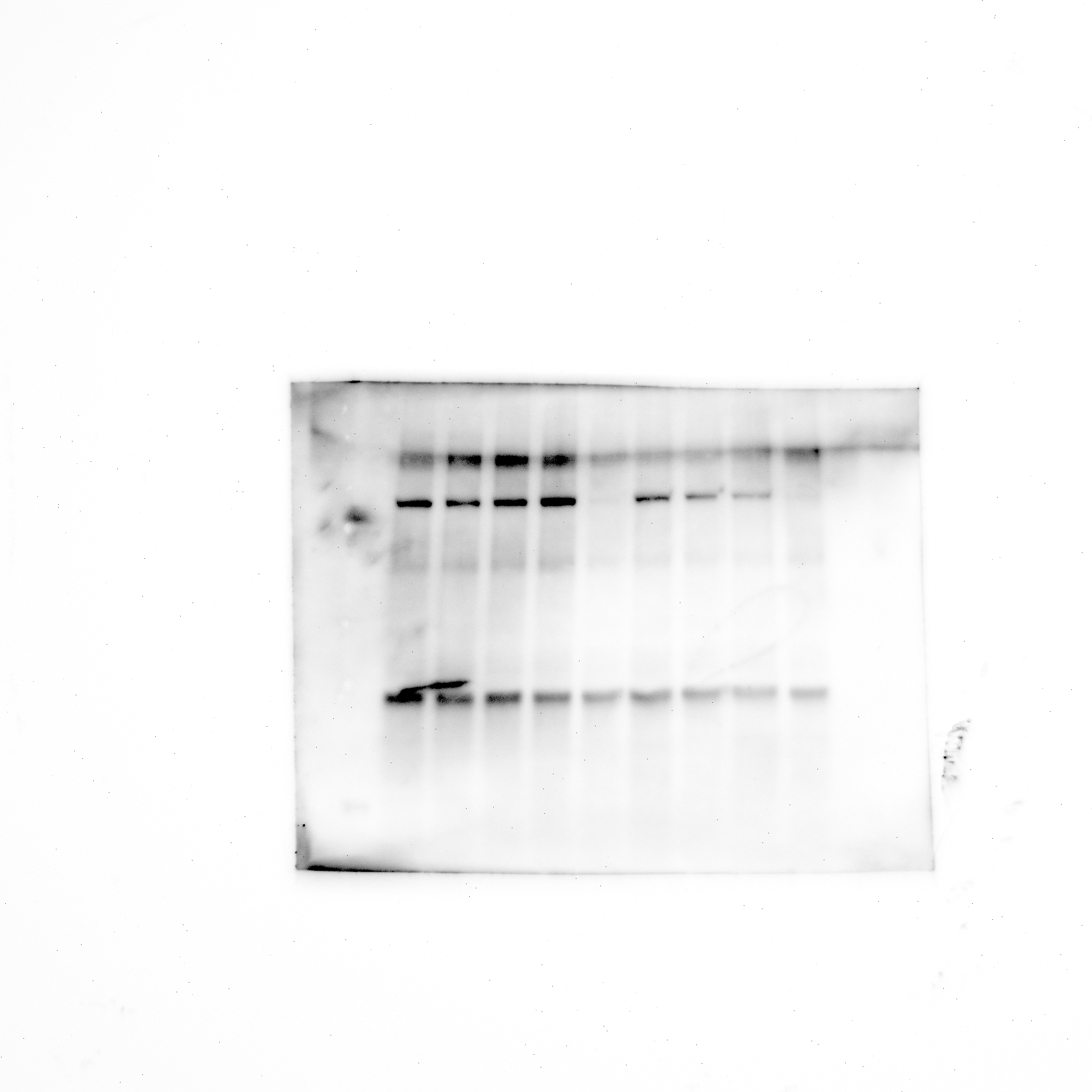

Supplement: Figure 5—source data 2. — Two close molecular markers are noted. Phospho-JAK2 at the predicted molecular weight is marked. The cropped region used for the main figure image is marked with a dotted box. This membrane was probed with the anti-phospho-JAK2 antibody. Top right – The same membrane as on the left, re-probed to visualize total-JAK2 signal. Bottom left – Original uncropped membrane from imager showing blue channel only as a black and white image. Two close molecular markers are noted. Phospho-STAT5b at the predicted molecular weight is marked. The cropped region used for the main figure image is marked with a dotted box. This membrane was probed with the anti-phospho-STAT5b antibody. Bottom right – The same membrane as on the bottom left, re-probed to visualize total-STAT5b signal. [file elife-90949-fig5-data2.zip › Figure 5-source data 2/Figure 5-source data 2 - original uncropped - immunoblot of male p-STAT5 after GH injection.tif]

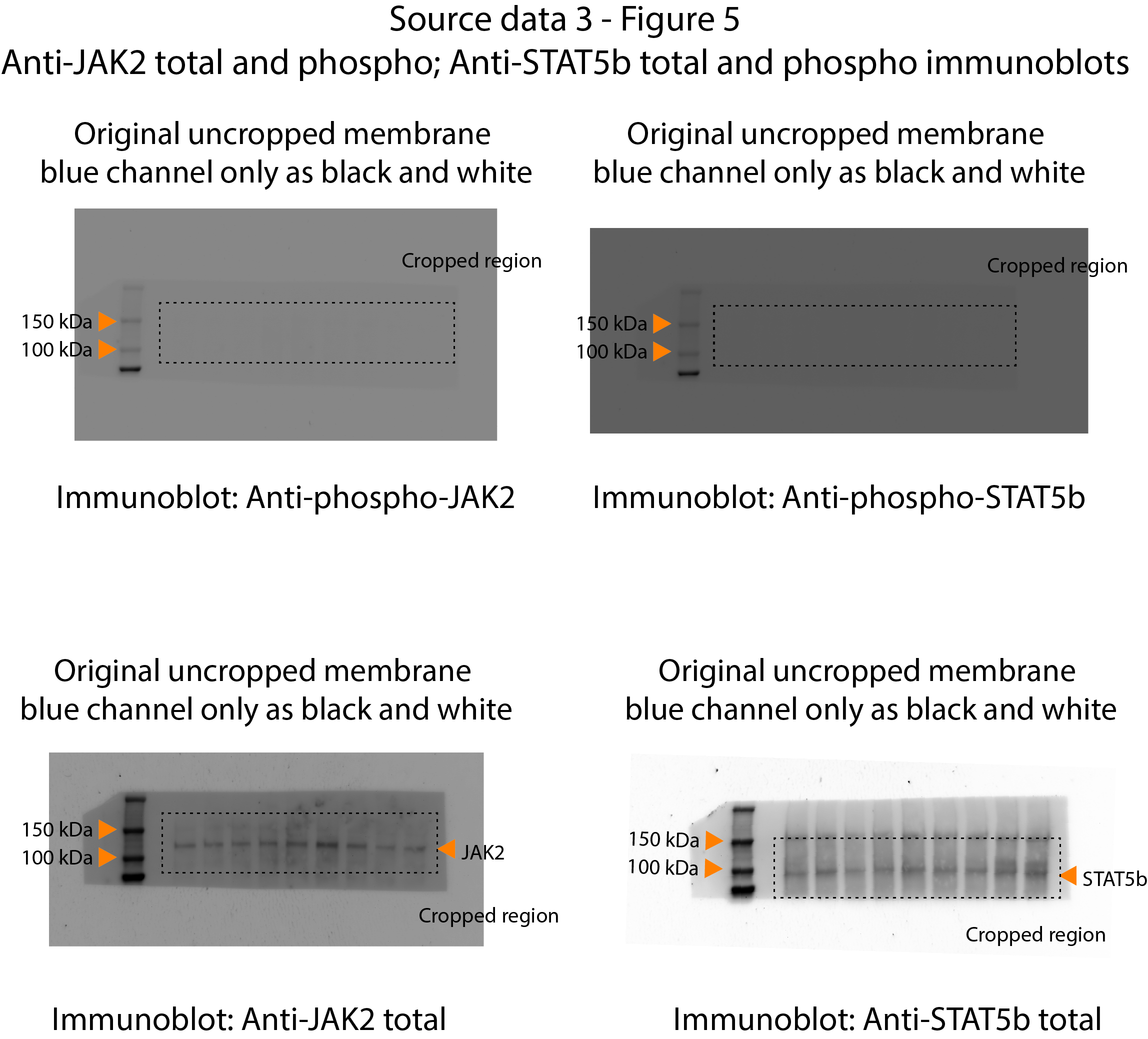

Supplement: Figure 5—source data 3. — Two close molecular markers are noted. Phospho-JAK2 at the predicted molecular weight is marked. The cropped region used for the main figure image is marked with a dotted box. This membrane was probed with the anti-phospho-JAK2 antibody. Bottom left – The same membrane as on the top left, re-probed to visualize total-JAK2 signal. Top right – Original uncropped membrane from imager showing blue channel only as a black and white image. Two close molecular markers are noted. Phospho-STAT5b at the predicted molecular weight is marked. The cropped region used for the main figure image is marked with a dotted box. This membrane was probed with the anti-phospho-STAT5 antibody. Bottom right – The same membrane as on the top right, re-probed to visualize total-STAT5b signal. [file elife-90949-fig5-data3.zip › Figure 5-source data 3.tif]

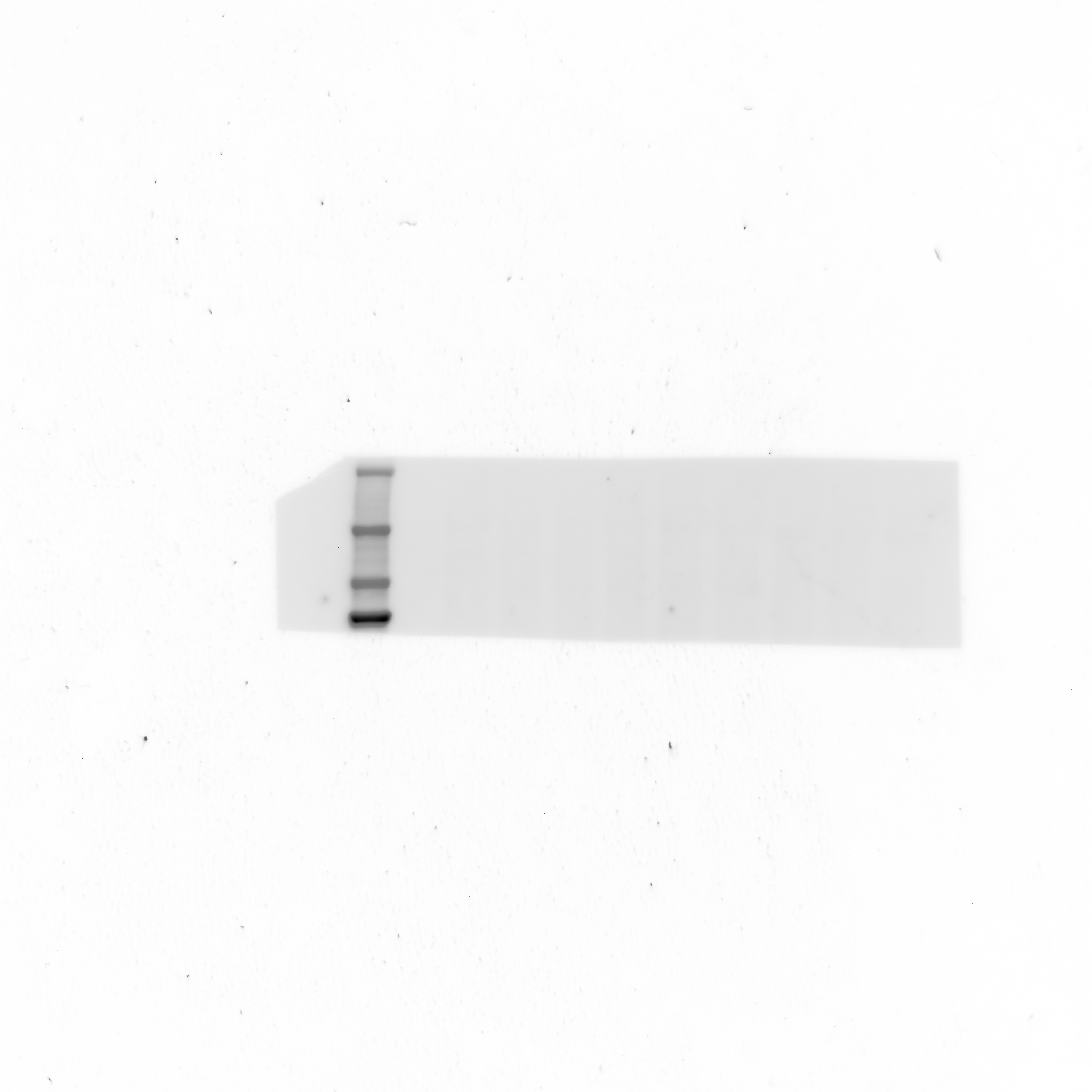

Supplement: Figure 5—source data 3. — Two close molecular markers are noted. Phospho-JAK2 at the predicted molecular weight is marked. The cropped region used for the main figure image is marked with a dotted box. This membrane was probed with the anti-phospho-JAK2 antibody. Bottom left – The same membrane as on the top left, re-probed to visualize total-JAK2 signal. Top right – Original uncropped membrane from imager showing blue channel only as a black and white image. Two close molecular markers are noted. Phospho-STAT5b at the predicted molecular weight is marked. The cropped region used for the main figure image is marked with a dotted box. This membrane was probed with the anti-phospho-STAT5 antibody. Bottom right – The same membrane as on the top right, re-probed to visualize total-STAT5b signal. [file elife-90949-fig5-data3.zip › Figure5-source data 3 - original uncropped membrane JAK2 phospho.tif]

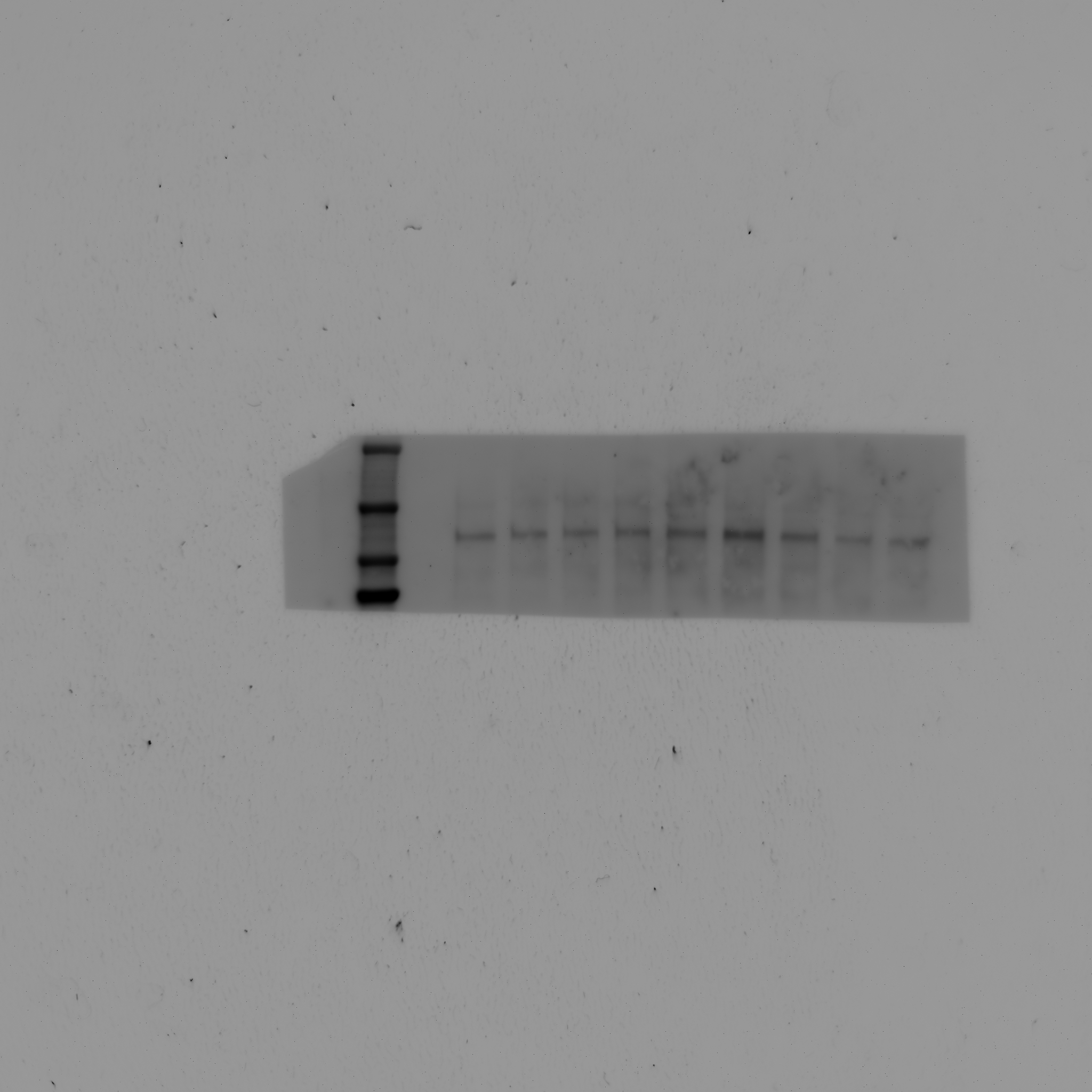

Supplement: Figure 5—source data 3. — Two close molecular markers are noted. Phospho-JAK2 at the predicted molecular weight is marked. The cropped region used for the main figure image is marked with a dotted box. This membrane was probed with the anti-phospho-JAK2 antibody. Bottom left – The same membrane as on the top left, re-probed to visualize total-JAK2 signal. Top right – Original uncropped membrane from imager showing blue channel only as a black and white image. Two close molecular markers are noted. Phospho-STAT5b at the predicted molecular weight is marked. The cropped region used for the main figure image is marked with a dotted box. This membrane was probed with the anti-phospho-STAT5 antibody. Bottom right – The same membrane as on the top right, re-probed to visualize total-STAT5b signal. [file elife-90949-fig5-data3.zip › Figure5-source data 3 - original uncropped membrane JAK2 Total.tif]

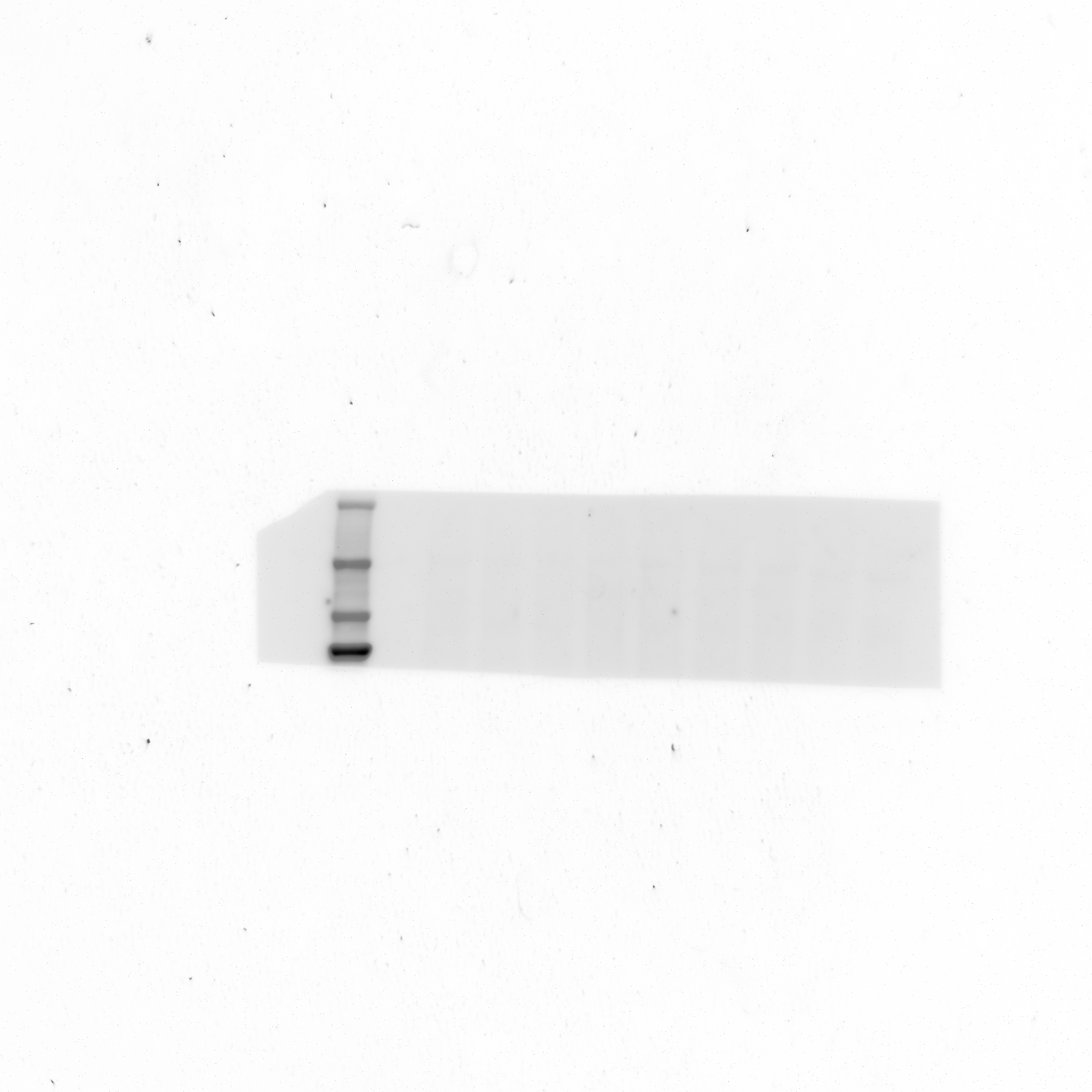

Supplement: Figure 5—source data 3. — Two close molecular markers are noted. Phospho-JAK2 at the predicted molecular weight is marked. The cropped region used for the main figure image is marked with a dotted box. This membrane was probed with the anti-phospho-JAK2 antibody. Bottom left – The same membrane as on the top left, re-probed to visualize total-JAK2 signal. Top right – Original uncropped membrane from imager showing blue channel only as a black and white image. Two close molecular markers are noted. Phospho-STAT5b at the predicted molecular weight is marked. The cropped region used for the main figure image is marked with a dotted box. This membrane was probed with the anti-phospho-STAT5 antibody. Bottom right – The same membrane as on the top right, re-probed to visualize total-STAT5b signal. [file elife-90949-fig5-data3.zip › Figure5-source data 3 - original uncropped membrane STAT5b phospho.tif]

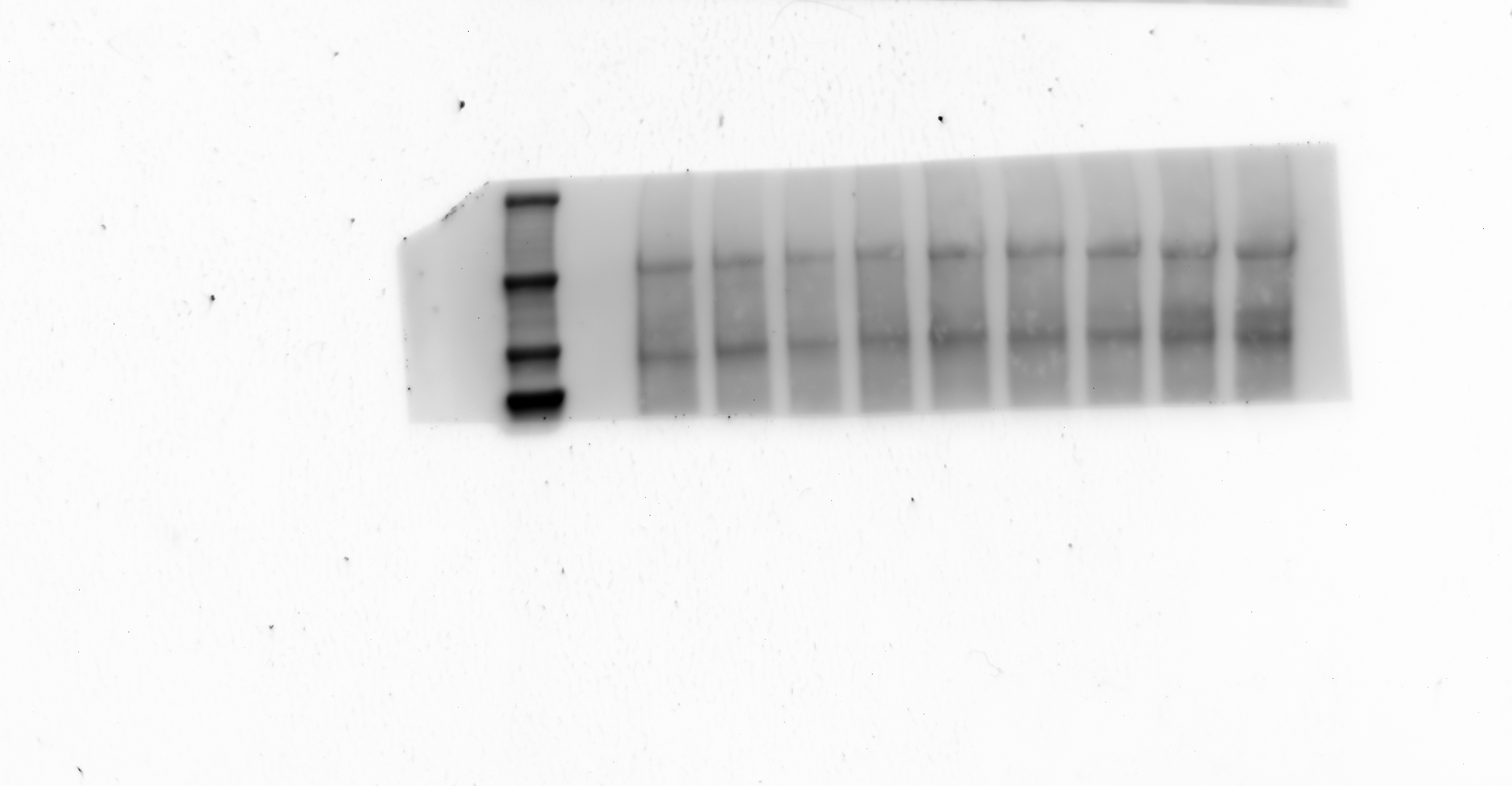

Supplement: Figure 5—source data 3. — Two close molecular markers are noted. Phospho-JAK2 at the predicted molecular weight is marked. The cropped region used for the main figure image is marked with a dotted box. This membrane was probed with the anti-phospho-JAK2 antibody. Bottom left – The same membrane as on the top left, re-probed to visualize total-JAK2 signal. Top right – Original uncropped membrane from imager showing blue channel only as a black and white image. Two close molecular markers are noted. Phospho-STAT5b at the predicted molecular weight is marked. The cropped region used for the main figure image is marked with a dotted box. This membrane was probed with the anti-phospho-STAT5 antibody. Bottom right – The same membrane as on the top right, re-probed to visualize total-STAT5b signal. [file elife-90949-fig5-data3.zip › Figure5-source data 3 - original uncropped membrane STAT5b Total.tif]
